# Supplementary material for: Liebig’s law of the minimum in the TGF-β/SMAD pathway
Source: PLoS Comput Biol. 2024 May 16;20(5):e1012072. doi: 10.1371/journal.pcbi.1012072 (PMC11135686; doi:10.1371/journal.pcbi.1012072)
Supplement: S1 File — (PDF) [file pcbi.1012072.s001.pdf]

Supporting Information for

Liebig's law of the minimum in the TGF-β/SMAD pathway

Yuchao Li, Difan Deng, Chris Tina Höfer, Jihye Kim, Won Do Heo, Quanbin Xu,

Xuedong Liu, Zhike Zi

Corresponding author: Zhike Zi

S1 File

Table of Contents

Appendix A: Supplementary Methods 1

1.1 The minimal mathematical model of the TGF-β pathway 1

1.2 The extended model of the TGF-β pathway 3

1.3 Parameter estimation for the extend model 4

1.4. Single cell model simulations 7

1.5 Measurement of TGF-β concentration in media 7

1.6 Cell surface protein biotinylation and internalization 8

Appendix B: Supplementary Tables 9

Table A: Observation of imbalanced *TGFBR1* and *TGFBR2* expression levels in HepG2 and RH30 cell lines across different RNA-Seq databases 9

Table B: Summary of the derived parameter values based on experimental data 10

Table C: Statistical analysis of the estimated parameters for the extended model 11

Table D: Initial conditions of the extended model 12

Table E: Ordinary differential equations of the extended model 13

Appendix C: Supplementary Figures 14

Fig A. The predicted response profile of TGF-β signaling activities (S_a) at steady state with different choice of parameter values in the minimal model. 14

Fig B. Measurement of protein abundance in HaCaT cells 15

Fig C. Measurement of TGFBR2 internalization rate constant in HaCaT cells 16

Fig D. The comparison of model simulation results and experimental data 17

Fig E. Profile likelihood analysis of estimated parameter values 18

Fig F. Computational modeling of the ratio of nuclear-to-cytoplasmic SMAD2 in the variation space of TGF-β receptors and SMAD2. 19

Fig G. Contour landscape of SMAD2 signaling responses in the variation space of TGF-β receptors and SMAD2 under different doses of TGF-β stimulations. 20

Fig H. TGFBR1 and TGFBR2 protein expression profiles in HaCaT, RH-30 and HepG2 cells 21

Fig I. Knockdown effect of TGFBR1 and TGFBR2 proteins using different concentrations of siRNAs in HaCaT, RH-30 and HepG2 cells 22

Fig J. Statistical analysis of TGF-β receptor fold-change effects leading to a 50% reduction in the P-Smad2 response compared to that in the non-targeting siRNA control group (EC50) during siRNA knockdown experiments. 23

Fig K. Expression of TGF-β targeted genes in responses to the knockdown of TGF-β receptors in HaCaT, RH30 and HepG2 cells 24

Fig L. Quantification of SMAD2 N2C fold change responses in HaCaT cells expressing similar levels of optoTGFBR1 and optoTGFBR2 25

Fig M. Western blot analysis of optoTGFBR1 and optoTGFBR2 protein expression in optoTGFBRs-HeLa cells transfected with optoTGFBR1 or optoTGFBR2 plasmids 26

References 27

Appendix A: Supplementary Methods

1.1 The minimal mathematical model of the TGF-β pathway

To investigate the signaling activity profiles of the TGF-β pathway in response to variations in TGF-β receptors, we developed a minimal model that captures the essential components of the pathway through two reactions. The first reaction represents the binding of the TGF-β ligand (L) to type I (R_1) and type II (R_2) TGF-β receptors, forming the ligand-receptor complex (LRC). The second reaction describes the phosphorylation and dephosphorylation of the SMAD protein (S_u: unphosphorylated Smad, S_p: phosphorylated Smad).

Following the approach used in published BMP pathway models [1, 2], we model the binding and unbinding of TGF-β ligand and its receptors as one-step reversible reaction with forward rate (k_f) and backward rate (k_b). We assumed that the phosphorylation of the SMAD protein is proportional to the activity of the ligand-receptor complex. In this minimal model, our focus is on the steady-state signaling activity of the TGF-β pathway. Additionally, we assumed the total amounts of receptors and SMAD proteins to remain constant (conservation of mass), following similar assumptions made in mathematical models of TGF-β and BMP pathway [1-3]. The two reactions can be summarized as:

$$L + R_1 + R_2 \underset{k_b}{\overset{k_f}{\rightleftharpoons}} LRC \tag{1}$$

$$S_u \underset{k_{depho}}{\overset{k_{pho}[LRC]}{\rightleftharpoons}} S_p \tag{2}$$

where S_u denotes unphosphorylated SMAD, S_p denotes phosphorylated SMAD.

Based on mass-action kinetics, we can formulate the following set of ordinary differential equations to describe the dynamics of these reactions:

$$\frac{d[R_1]}{dt} = k_b[LRC] - k_f[L][R_1][R_2] \tag{3}$$

$$\frac{d[R_2]}{dt} = k_b[LRC] - k_f[L][R_1][R_2] \tag{4}$$

$$\frac{d[LRC]}{dt} = k_f[L][R_1][R_2] - k_b[LRC] \tag{5}$$

$$\frac{d[pS]}{dt} = k_{pho}[S_u][LRC] - k_{depho}[S_p] \tag{6}$$

With the mass of conservation, we obtain the following constraints:

$$R_1^t = [R_1] + [LRC] \quad (7)$$

$$R_2^t = [R_2] + [LRC] \quad (8)$$

$$S^t = [S_u] + [S_p] \quad (9)$$

where the variables with superscript 't' denote the total of the respective species with mass conservation.

Furthermore, in typical experimental setups, the TGF-β ligand is prepared in a much larger volume of media compared to the volume of cells, resulting in a scenario where there are significantly more ligand molecules than receptors. In such cases, the concentration of the TGF-β ligand remains constant.

$$[L] = L^t \quad (10)$$

By plugging equation 9 to equation 6 and scaling it to S^t, equation 6 can be rewritten as:

$$\frac{dS_a}{dt} = k_{pho}(1 - S_a)[LRC] - k_{depho} \cdot S_a \quad (11)$$

where S_a represents the TGF-β signaling activity, which is the percentage of total SMAD proteins that are actively phosphorylated.

We next focused on the TGF-β signaling activity (S_a) at steady state after ligand stimulation, which can be solved based on the following algebraic equations:

$$k_b[LRC^{ss}] - k_f[L^{ss}][R_1^{ss}][R_2^{ss}] = 0 \quad (12)$$

$$k_{pho}(1 - S_a^{ss})[LRC^{ss}] - k_{depho} \cdot S_a^{ss} = 0 \quad (13)$$

Plugging equations 7-9 to equations 12 and 13, we can derive the steady state of the TGF-β signaling activity (S_a^{ss}) as the following:

$$LRC^{ss} = \frac{\left(1+\frac{k_f}{k_b}L^tR_1^tR_2^t\right) - \sqrt{\left(1+\frac{k_f}{k_b}L^tR_1^tR_2^t\right)^2 - 4\left(\frac{k_f}{k_b}L^t\right)^2 R_1^tR_2^t}}{2\frac{kf}{kb}L^t} \quad (14)$$

$$S_a^{ss} = \frac{k_{pho}[LRC^{ss}]}{k_{pho}[LRC^{ss}] + k_{depho}} \quad (15)$$

Defining the following parameters

$$K_1 = \frac{k_f}{k_b} \quad (16)$$

$$K_2 = \frac{k_{pho}}{k_{depho}} \tag{17}$$

$$\alpha = 1 + K_1 L^t R_1^t R_2^t \tag{18}$$

$$\beta = 4\left(K_1 L^t\right)^2 R_1^t R_2^t \tag{19}$$

Equation 14 and 15 can be rewritten as

$$[LRC^{ss}] = \frac{\alpha - \sqrt{\alpha^2 - \beta}}{2K_1 L^t} \tag{20}$$

$$S_a^{ss} = \frac{K_2[LRC^{ss}]}{K_2[LRC^{ss}]+1} = \frac{K_2\left(\alpha - \sqrt{\alpha^2-\beta}\right)}{K_2\left(\alpha - \sqrt{\alpha^2-\beta}\right) + 2K_1 L^t} \tag{21}$$

With equation 18, 19 and 21, we can plot the response profiles of the TGF-β signaling activity at steady state (S_a^{ss}) with the combinations of different R1 and R2 values. The initial abundance (N_X, molecules per cell) and the initial concentration (X^0) of specie X can be exchanged with the following equation:

$$N_X = N_A V_{cell} X^0 \tag{22}$$

where N_A is Avogadro number (6.02×1023). V_{cell} is set to be 3.3 pL (3.3×10-12 L) according to previous reports [4]. It's worth noting that the choice of V_{cell} value does not change the response profiles shown in Fig 3 as cell volume is just a scaling factor for the conversion between specie concentration and abundance.

1.2 The extended model of the TGF-β pathway

We next developed an extended mathematical model based on our previously published TGF-β model [5]. Compared to the minimal model, the extended model includes additional TGF-β signaling processes: (1) receptor production, degradation, endocytosis and activation; (2) SMAD2 nucleocytoplasmic shuttling, phosphorylation and dephosphorylation; (3) negative feedback on the degradation of ligand receptor complex; (4) reversible binding of TGF-β to the cell surface. In the extended model, we used a linear chain of 2 variables (z1, z2) with linear differential equations to model the time delay between nuclear SMAD2 activity (PSmad2n) and the negative feedback on the degradation of ligand-receptor complex.

The extended model consists of 13 species and 22 reactions, characterized by 20 kinetic parameters. Details regarding the initial conditions, parameter values, and equations can be

found in Table B-E in S1 File. To determine the initial condition of the extended model, we measured the abundance of SMAD2 and TGF-β receptors in the HaCaT cell line (as described in section 1.2). Additionally, we derived 11 kinetic parameters from experimental datasets, including our own and those from other studies. The remaining 9 unknown parameter values were estimated by fitting the model to 102 experimental data points obtained from HaCaT cells exposed to different TGF-β stimulation conditions. Notably, parameter identifiability analysis confirms the identifiability of these parameters (see section 1.3.5 for more details on the estimation of unknown parameters).

1.3 Parameter estimation for the extend model

1.3.1 The abundance of SMAD2 and TGF-β receptors

To determine the abundance of SMAD2 and TGF-β type II receptor (TGFBR2, referred to as T2R in the extended model), we performed quantitative immunoblotting on HaCaT cells. This technique allowed us to estimate the absolute protein levels based on a standard curve signal generated from recombinant protein standards. The detailed procedure is described in a published protocol [6]. The measured abundances of SMAD2 and TGFBR2 proteins in HaCaT are 1.54e5 and 2.06e4 molecules/cell, respectively (Fig B in S1 File).

According to the Human Protein Atlas database, the expression of TGFBR1 RNA in HaCaT cells is similar to that of TGFBR2. In the extended model, we set the protein abundance of TGFBR1 to be the same as TGFBR2 (2.06e4 molecules/cell), which aligns with a previously reported value [7].

1.3.2 TGF-β receptor internalization and recycling rate constants

We measured the internalization rate of TGFBR2 using a reversible biotinylation method [8]. We next fitted the relative change of surface receptor protein over time with an exponential decay function and successfully measured the internalization rate constant of TGFBR2: $k_i = 0.022\ min^{-1} = 1.32\ h^{-1}$ (Fig C in S1 File).

It is known that most of TGF-β receptors are internalized in endosomes and about 10% of TGF-β receptors locate at cell surface [9]. Here we set the recycling rate is 1/9 of the internalization rate, $k_r = k_i/9 = 0.147\ h^{-1}$, this will result in about 10% receptors at cell surface in our model.

1.3.3 TGF-β receptor production and degradation rate constants

We measured the half-lives of TGF-β receptors and SMAD2 proteins in HaCaT cells by cycloheximide chase assays [10]. The measured half-lives for TGFBR1, TGFBR2 and SMAD2 are 6.6 h, 3.67h, >24 h, respectively. The SMAD2 protein is stable before and after TGF-β stimulation. It is reasonable to assume the total amount of SMAD2 protein is constant in the model.

By rescaling the distribution of internalized receptors in endosomes, we can derive the degradation rate constants for T1R: $k_{deg}^{T1R} = 0.117\ h^{-1}$ $(\frac{-ln0.5}{6.6h\times 0.9})$, for T2R: $k_{deg}^{T2R} = 0.21\ h^{-1}$ $(\frac{-ln0.5}{3.67h\times 0.9})$. Since TGF-β ligand receptor complexes have similar distribution and half-life as the T1R receptor at resting state [9], we set $k_{deg}^{LCR} = k_{deg}^{T1R} = 0.117\ h^{-1}$.

The initial conditions of TGF-β receptors are determined by the receptor production, internalization, recycling and degradation rate constants [5, 11]. At resting state, we derive the following equations for TGF-β receptors at cell surface (*T1Rs*, *T2Rs*) and in cell cytosol (*T1Rc*, *T2Rc*):

$$[T1R_s]_0 = \frac{k_{prod}^{T1R} \cdot kr}{k_{deg}^{T1R} \cdot ki} \quad (23)$$

$$[T1R_c]_0 = \frac{k_{prod}^{T1R}}{k_{deg}^{T1R}} \quad (24)$$

$$N_{T1R} = V_{cyt}([T1R_s]_0 + [T1R_c]_0)N_A \quad (25)$$

$$[T2R_s]_0 = \frac{k_{prod}^{T2R} \cdot kr}{k_{deg}^{T2R} \cdot ki} \quad (26)$$

$$[T2R_c]_0 = \frac{k_{prod}^{T2R}}{k_{deg}^{T2R}} \quad (27)$$

$$N_{T2R} = V_{cyt}([T2R_s]_0 + [T2R_c]_0)N_A \quad (28)$$

where N_A is Avogadro number (6.02×10^{23}), N_{T1R} and N_{T2R} are the abundances of TGFBR1 and TGFBR2 in molecules/cell, respectively.

The volume of cytoplasm (V_{cyt}) and nucleus (V_{nuc}) are set as 2.3 pL (2.3×10^{-12} L) and 1 pL (1×10^{-12} L), respectively according to previous reports [4]. Solving equation 1-6, we can derive the initial conditions for T1R and T2R at cell surface and in cytosol with the following equations:

$$[T1R_s]_0 = \frac{N_{T1R}}{V_{cyt}N_A} \cdot \frac{kr}{ki+kr} = 1.49 \times 10^{-9}\, M = 1.49\, nM \quad (29)$$

$$[T1R_c]_0 = \frac{N_{T1R}}{V_{cyt}N_A} \cdot \frac{ki}{ki+kr} = 13.4 \times 10^{-9}\, M = 13.4\, nM \quad (30)$$

$$[T2R_s]_0 = \frac{N_{T2R}}{V_{cyt}N_A} \cdot \frac{kr}{ki+kr} = 1.49 \times 10^{-9}\, M = 1.49\, nM \quad (31)$$

$$[T2R_c]_0 = \frac{N_{T2R}}{V_{cyt}N_A} \cdot \frac{ki}{ki+kr} = 13.4 \times 10^{-9}\, M = 13.4\, nM \quad (32)$$

Based on the equations 24, 30 and equations 27, 32, we derived the corresponding production rate constant for T1R ant T2R:

$$k_{prod}^{T1R} = k_{deg}^{T1R}[T1R_c]_0 = 0.117 \times 13.4 = 1.57\, nM/h \quad (33)$$

$$k_{prod}^{T2R} = k_{deg}^{T2R}[T2R_c]_0 = 0.21 \times 13.4 = 2.81\, nM/h \quad (34)$$

1.3.4 Initial conditions for cytosol and nuclear SMAD2

SMAD2 nuclear import and export rate constants has been experimentally measured [4, 12]: $k_{imp}^{Smad2} = 0.156\, min^{-1} = 9.36\, h^{-1}$, $k_{exp}^{Smad2} = 0.763\, min^{-1} = 45.8\, h^{-1}$. We set the distribution of SMAD2 in cytoplasm and nucleus in steady state before TGF-β stimulation with the following equations

$$[Smad2_c]_0 = \frac{N_{Smad2}}{V_{cyt}N_A} \cdot \frac{k_{exp}^{Smad2}}{k_{imp}^{Smad2}+k_{exp}^{Smad2}} = 92.4\, nM \quad (35)$$

$$[Smad2_n]_0 = \frac{N_{Smad2}}{V_{nuc}N_A} \cdot \frac{k_{imp}^{Smad2}}{k_{imp}^{Smad2}+k_{exp}^{Smad2}} = 43.4\, nM \quad (36)$$

where N_A is Avogadro number (6.02×10^{23}), N_{SMAD2} is the abundances of SMAD2 in molecules/cell.

1.3.5 Estimation of the unknown parameters

We measured or derived most parameter values in the refined models based on experimental data. The detailed values for these parameters are shown in Table B in S1 File. In the refined model, there were still some parameters that cannot be measured or derived directly from experimental data. For estimation of these unknown parameters, we used a parallel parameter estimation tool SBML-PET-MPI [13], which includes a global optimization algorithm (SRES) using least squares to minimize the sum of squares of differences between model simulations and the corresponding experimental datasets. We estimated the values of 9 parameters in the

model with 102 experimental data points collected from HaCaT cells with different TGF-β stimulation conditions, which includes.

(1) Time course of medium TGF-β (TGFb_ex in the model) and SMAD2 phosphorylation (P-SMAD2) levels in response to 10 pM, 100 pM and 400pM TGF-β stimulations.

(2) Time course of cytosol and nuclear SMAD2 in response to 100 pM TGF-β stimulation.

(3) Relative P-SMAD2 level at 45 min in responses to different doses of TGF-β stimulations.

After parameter optimization, the refined model can reproduce various experimental data with different doses of TGF-β stimulations (Fig D in S1 File). To check the identifiability of the estimated parameters in the model, we performed profile likelihood analysis [14] and calculate 95% confidence intervals for the estimated parameters in the model. The profile likelihood analysis indicate that the estimated parameters are practically identifiable with the experimental datasets used for parameter estimation.

1.4. Single cell model simulations

To simulate the signaling responses in single cells, we use the same model as the population model except that the abundances of TGFBR1 (N_{T1R}), TGFBR2 (N_{T2R}) and SMAD2 (N_{Smad2}) are randomly generated from log-normal distributions with a mean of the corresponding reference value in the population model and a CV of 0.1. For each single cell model, we adjust the receptor production parameter values, while keep other kinetic parameter values fixed. The production rate constants for TGFBR1 (kprd_T1R) and TGFBR2 (kprd_T2R) are set according to equation 33 and equation 34, respectively. The initial conditions for surface and cytosol receptors can be derived with equation 29-32. The initial concentrations of cytosol SMAD2 and nuclear SMAD2 were calculated based on equation 35-36 accordingly. For the variation of negative feedback regulation (NFR), we randomly generate a value for k_NFR from a log-normal distribution with a mean of the corresponding reference value in the population model and a CV of 0.1. For each case, we performed 5000 single cell model simulations and the correlation analysis results were calculated with Pearson correlation coefficients.

1.5 Measurement of TGF-β concentration in media

Medium supernatants were collected from the culture of HaCaT cells at different time points after TGF-β1 (R&D Systems, Catalog Number: 240-B-002) stimulations. Medium TGF-β1 concentrations were measured using a TGF beta 1 ELISA kit (invitrogen, Catalog Number: 88-8350) following the manufacturer's instructions.

1.6 Cell surface protein biotinylation and internalization

The internalization rate of TGFBR2 was determined using a reversible biotinylation method [8] with a cell surface protein isolation kit (Thermo Scientific Pierce, Catalog Number: 89881). Cell surface proteins were labeled with biotinylation reagent (sulfo-NHS-SS-biotin) at a low temperature (4 °C). A portion of the cells were shifted to permissive conditions for trafficking at 37 °C, allowing the biotinylated receptor proteins to internalize. Another set of cells was maintained at the low temperature as controls for total surface protein at time=0 and for stripping control. After different time of internalization, cells were moved back to low temperature to stop internalization and the residual surface biotin was stripped off with a reducing agent, which cleaves the disulfide-coupled biotin. The internalized receptor proteins with biotin were protected and they were not stripped. After cell lysis, biotinylated proteins were pulled down by streptavidin affinity chromatography according to the manufacturer's protocol and they were measured using quantitative immunoblotting. By subtracting the internalized receptor protein from the total surface protein control at time=0, the remaining surface receptor protein at different time points was determined. The dynamics of the remaining surface TGFBR2 were fitted with an exponential decay function to estimate the internalization rate constant.

Appendix B: Supplementary Tables

Table A: Observation of imbalanced *TGFBR1* and *TGFBR2* expression levels in HepG2 and RH30 cell lines across different RNA-Seq databases

Cell Line	TGFBR1	TGFBR2	TGFBR1-to-TGFBR2 ratio	Data Source
HepG2	2.94	48.46	0.06	ENCODE
HepG2	5.05	58.49	0.09	Cell Model Passports (Sanger)
HepG2	15.9	114	0.14	Human Protein Atlas
RH30	32.96	9.51	3.47	Cell Model Passports (Sanger)
RH30	171.4	40.6	4.22	Human Protein Atlas

Note: RNA expression levels are presented in normalized transcripts per million (nTPM) for data from Human Protein Atlas and in fragments per kilobase per million mapped fragments (FPKM) for data from other databases.

Table B: Summary of the derived parameter values based on experimental data

Parameter	Value and Unit	Annotation	Reference
ki	1.32 h^{-1}	receptor internalization rate constant	this work
kr	0.147 h^{-1}	receptor recycling rate constant	this work
kprod_T1R	1.57 nM/h	TGFBR1 production rate constant	this work
kprod_T2R	2.81 nM/h	TGFBR2 production rate constant	this work
kdeg_T1R	0.117 h^{-1}	constitutive degradation rate constant for TGFBR1	this work
kdeg_T2R	0.21 h^{-1}	constitutive degradation rate constant for TGFBR2	this work
kdeg_LRC	0.117 h^{-1}	constitutive degradation rate constant for ligand receptor complex (LRC)	this work
kdeg1_TGFb	20.8 h^{-1}	constitutive degradation rate constant for internalized TGF-β	[15, 16]
kimp_Smad2	9.36 h^{-1}	SMAD2 nuclear import rate constant	[4, 5]
kexp_Smad2	45.8 h^{-1}	SMAD2 nuclear export rate constant	[4, 5]
kimp_PSmad2	53.3 h^{-1}	P-SMAD2 nuclear import rate constant	[4]
Vcyt	2.3e^{-12} l	Cytoplasm volume	[4]
Vnuc	1e^{-12} l	Nucleus volume	[4]
Vmed	2e^{-9} l	Extracellular space volume	[5]

Note: The derived parameters were rounded with 3 effective digits

Table C: Statistical analysis of the estimated parameters for the extended model

Parameter (Unit)	Best fit	95% confidence interval	Annotation
ka_LRC ($nM^{-2}h^{-1}$)	6.57019e+02	(6.25195e+02; 6.89870e+02)	Activation rate constant for ligand-receptor complex
kdiss_LRC (h^{-1})	1.74990e+00	(1.68428e+00; 1.82099e+00)	Dissociation rate constant for ligand-receptor complex
k_NFR ($nM^{-1}h^{-1}$)	6.04124e-02	(5.89965e-02; 6.18755e-02)	Rate constant for negative feedback regulation
kdeg2_TGFb (h^{-1})	1.38784e-01	(1.25339e-01; 1.52663e-01)	Degradation of TGF-β through non-specific binding
kpho_Smad2 ($nM^{-1}h^{-1}$)	1.68603e+00	(1.64520e+00; 1.72687e+00)	SMAD2 phosphorylation rate constant
kdepho_Smad2 (h^{-1})	1.94430e+00	(1.89266e+00; 1.99747e+00)	SMAD2 dephosphorylation rate constant
kon_ns (h^{-1})	3.18848e-01	(3.09881e-01; 3.27816e-01)	Association rate constant for non-specific binding of TGF-β
koff_ns (h^{-1})	1.00995e+00	(9.73658e-01; 1.04783e+00)	Dissociation rate constant for non-specific binding of TGF-β
tau (h)	2.00973e+00	(1.95320e+00; 2.06782e+00)	Delay time between negative feedback regulation and P-Smad2n

Note: The estimated parameters were rounded with 6 effective digits.

Table D: Initial conditions of the extended model

Species	Initial Conditions (Unit: nM)	Annotation
TGFb_ex	to be specified	TGF-β concentration in the medium
T1Rs	1.49	type I receptor at plasma membrane (cell surface)
T1Rc	13.4	type I receptor in early endosome
T2Rs	1.49	type II receptor at plasma membrane (cell surface)
T2Rc	13.4	type II receptor in early endosome
LRCs	0	LRC at plasma membrane (cell surface)
LRCc	0	LRC in early endosome
Smad2c	92.4	SMAD2 in cytoplasm
Smad2n	43.4	SMAD2 in nucleus
PSmad2c	0	phosphorylated SMAD2 monomer in cytoplasm
PSmad2n	0	phosphorylated SMAD2 monomer in nucleus
TGFb_c	0	TGF-β in early endosome
TGFb_ns	0	non-specific bound TGF-β
z1	0	linear chain variable for the delay between PSmad2n and negative feedback regulation (NFR)
z2	0	linear chain variable for the delay between PSmad2n and negative feedback regulation (NFR)

Table E: Ordinary differential equations of the extended model

```
d[TGFb_ex]/dt = koff_ns*[TGFb_c] - kon_ns*[TGFb_ex] -
                Vcyt*kass_LRC*[TGFb_ex]*[T1Rs]*[T2Rs]/Vmed
d[T1Rs]/dt = kr*[T1Rc] - ki*[T1Rs] - kass_LRC*[TGFb_ex]*[T1Rs]*[T2Rs]
d[T1Rc]/dt = kprd_T1R - kdeg_T1R*[T1Rc] + kdiss_LRC*[LRCc] + ki*[T1Rs] - kr*[T1Rc]
d[T2Rs]/dt = kr*[T2Rc] - ki*[T2Rs] - kass_LRC*[TGFb_ex]*[T1Rs]*[T2Rs]
d[T2Rc]/dt = kprd_T2R - kdeg_T2R*[T2Rc] + kdiss_LRC*[LRCc] + ki*[T2Rs] - kr*[T2Rc]
d[LRCs]/dt = kass_LRC*[TGFb_ex]*[T1Rs]*[T2Rs] - k_NFR*[z2]*[LRCs] - ki*[LRCs]
d[LRCc]/dt = ki*[LRCs] - kdiss_LRC*[LRCc] - kdeg_LRC*[LRCc]
d[Smad2c]/dt = Vnuc*kexp_Smad2*[Smad2n]/Vcyt - kpho_Smad2*([LRCs]+[LRCc])*[Smad2c] -
               kimp_Smad2*[Smad2c]
d[Smad2n]/dt = Vcyt*kimp_Smad2*[Smad2c]/Vnuc + kdepho_Smad2*[PSmad2n] - kexp_Smad2*[Smad2n]
d[PSmad2c]/dt = kpho_Smad2*([LRCs]+[LRCc])*[Smad2c] - kimp_PSmad2*[PSmad2c]
d[PSmad2n]/dt = Vcyt*kimp_PSmad2*[PSmad2c]/Vnuc - kdepho_Smad2*[PSmad2n]
d[TGFb_c]/dt = kon_ns*[TGFb_ex] - koff_ns*[TGFb_c] - kdeg2_TGFb*[TGFb_c]
d[TGFb_ns]/dt = kdiss_LRC*[LRCc] - kdeg1_TGFb*[TGFb_ns]
d[z1]/dt = (2*([PSmad2n] - [z1]))/tau
d[z2]/dt = (2*([z1] - [z2]))/tau
```

Appendix C: Supplementary Figures

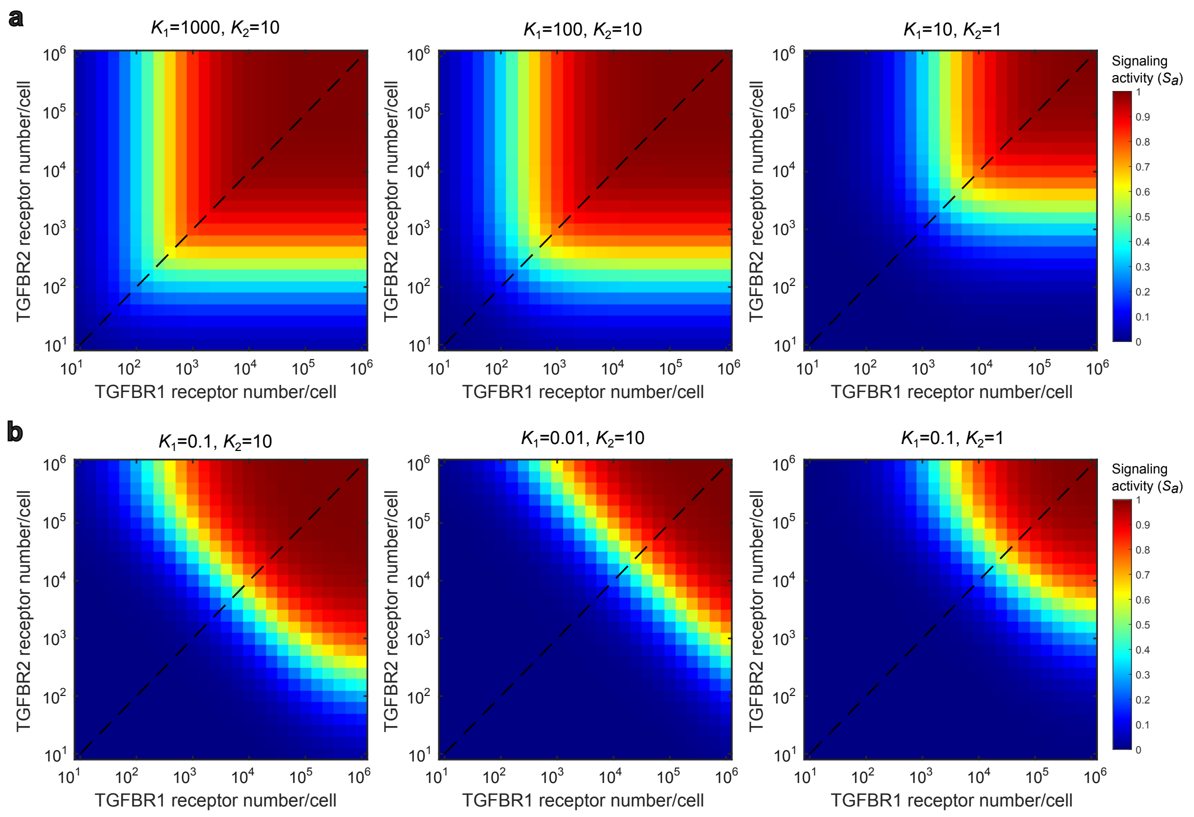

Fig A. The predicted response profile of TGF-β signaling activities (S_a) at steady state with different choice of parameter values in the minimal model.

(a) Simulations with high binding affinity values for K_1 ($K_1 = 1000, 100, 10$).

(b) Simulation with low binding affinity values for K_1 ($K_1 = 0.1, 0.01$).

The ligand concentration L is set to be 0.1 nM (100 pM) in all the simulations.

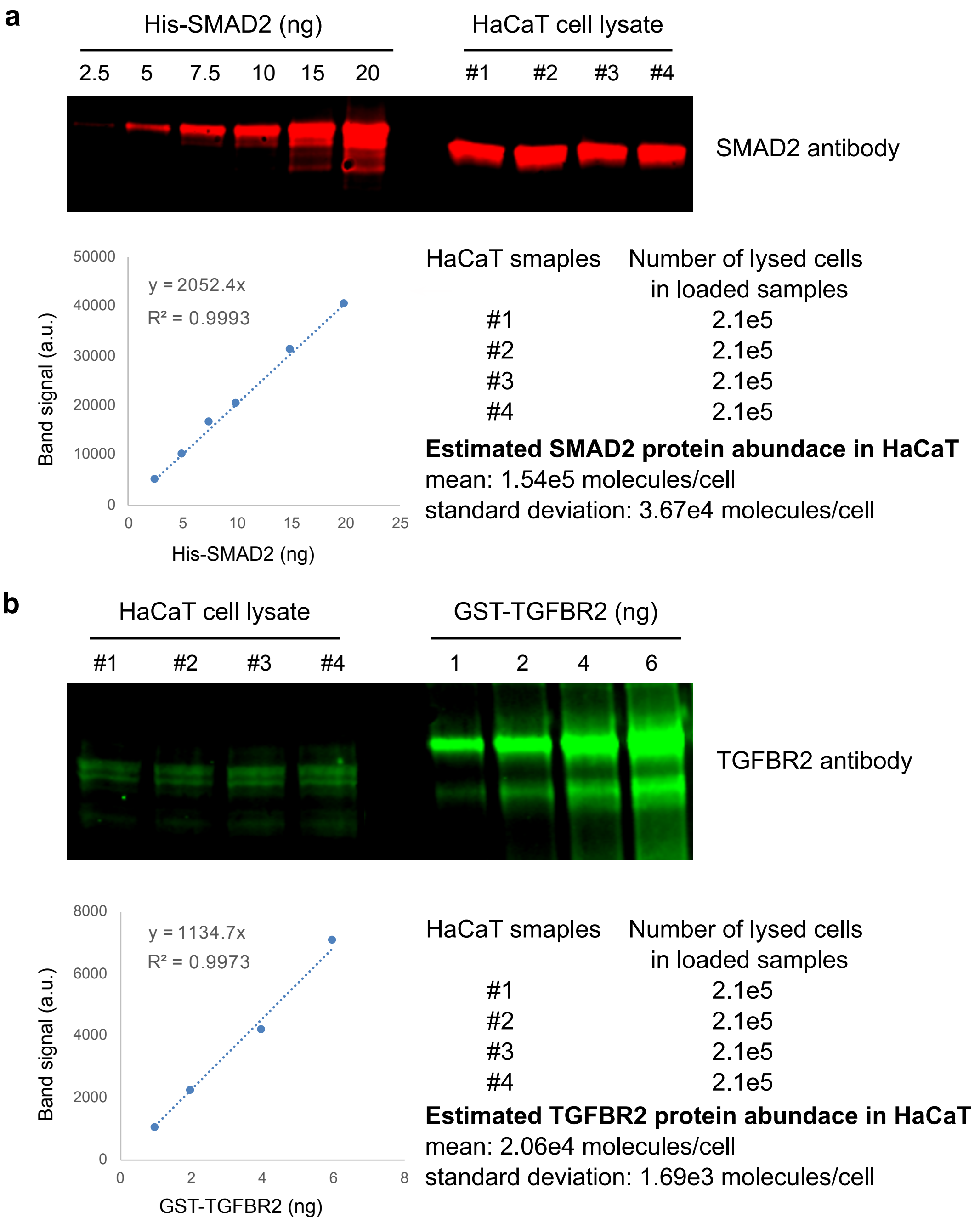

Fig B. Measurement of protein abundance in HaCaT cells

Protein abundances were determined by quantitative immunoblotting with a series of recombinant protein standard.

(a) Estimation of SMAD2 abundance in HaCaT cells.

(b) Estimation of TGFBR2 abundance in HaCaT cells.

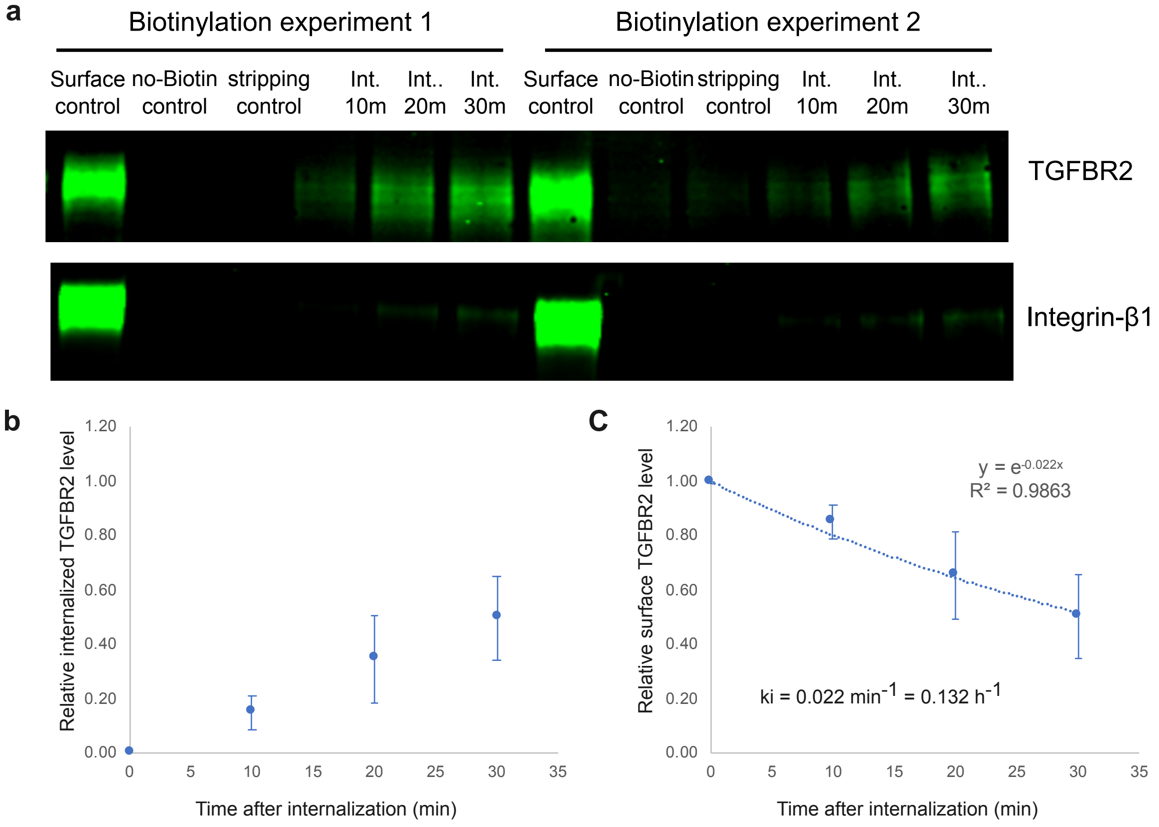

Fig C. Measurement of TGFBR2 internalization rate constant in HaCaT cells

(a) Immunoblotting of the biotinylated protein samples. The biotin labeling procedure is described in the Supplementary Methods 1.6 section. Integrin-β1 is a cell surface protein marker.

(b) The kinetics of the internalized TGFBR2 were quantified from Western blots. The mean values and standard deviations from 4 experiments are shown.

(c) The kinetics of the remaining surface TGFBR2 after internalization. The mean values and standard deviations from 4 experiments are shown. The data points were fitted to an exponential decay function and the internalization rate constant (ki) was estimated.

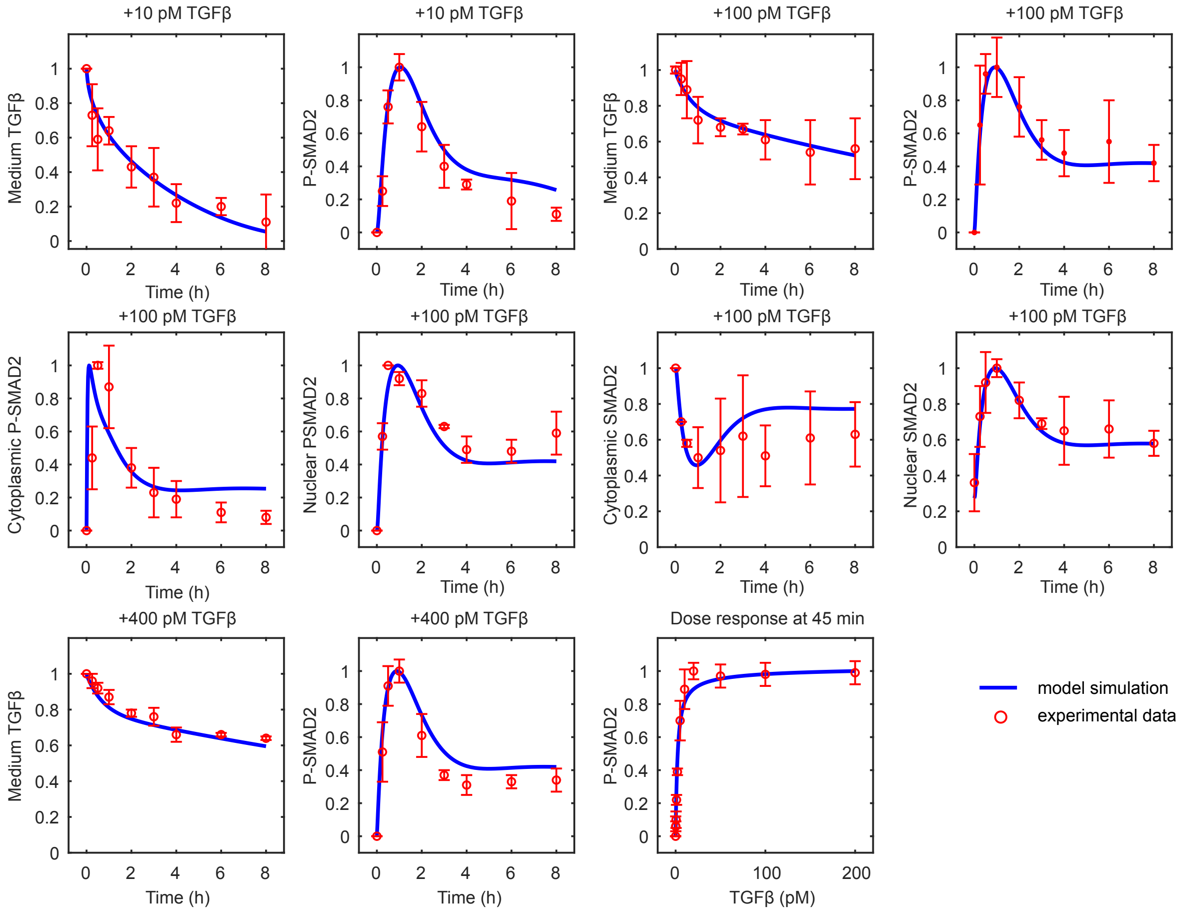

Fig D. The comparison of model simulation results and experimental data

For comparison, the model simulation results and experimental data are normalized their maximum values in the corresponding data sets. The mean values and standard deviations of the normalized data from 3-5 experiments are shown.

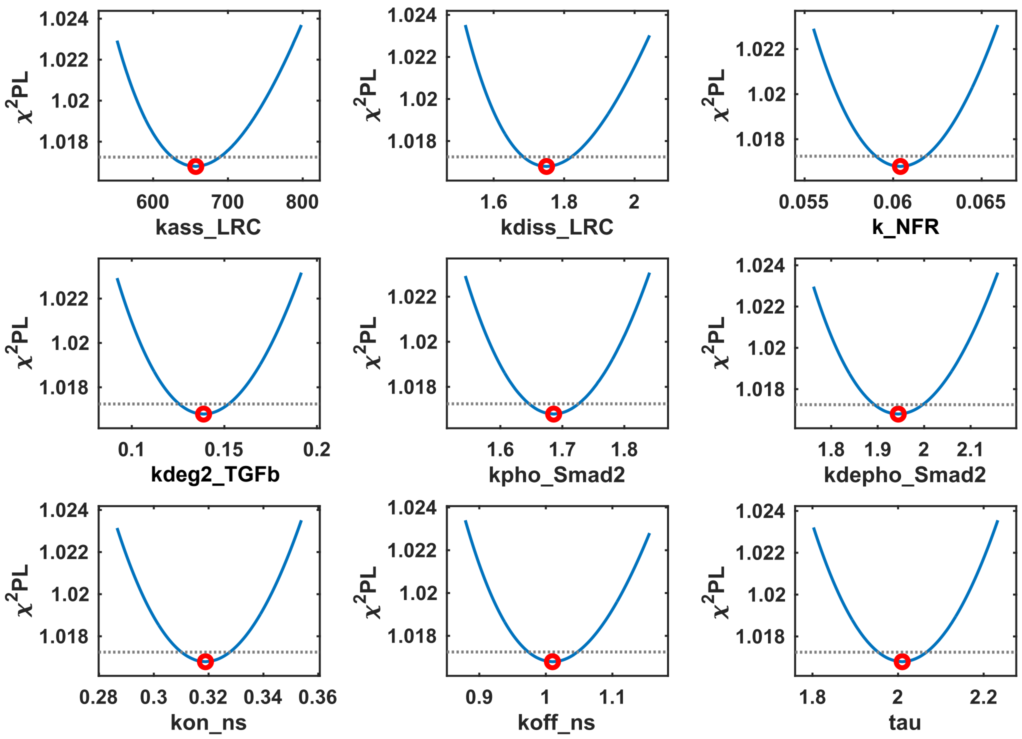

Fig E. Profile likelihood analysis of estimated parameter values

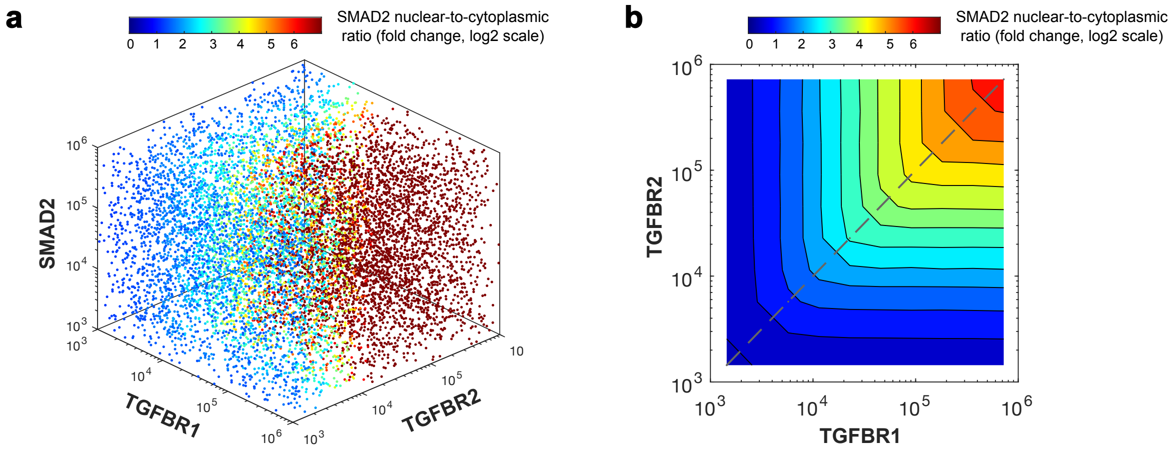

Fig F. Computational modeling of the ratio of nuclear-to-cytoplasmic SMAD2 in the variation space of TGF-β receptors and SMAD2.

(a) Model predictions for the fold change in the ratio of nuclear-to-cytoplasmic SMAD2 (N2C) in the space of different amounts of TGFBR1, TGFBR2, and SMAD2 . Each point indicates a random set of protein amounts in the space. Colors indicate the fold change in the SMAD2 N2C ratio.

(b) Contour landscape of the fold change in SMAD2 N2C according to combinations of different amounts of TGFBR1 and TGFBR2. The dashed line indicates relationship between the fold change in SMAD2 N2C and the receptors when equal amounts of each receptor are present.

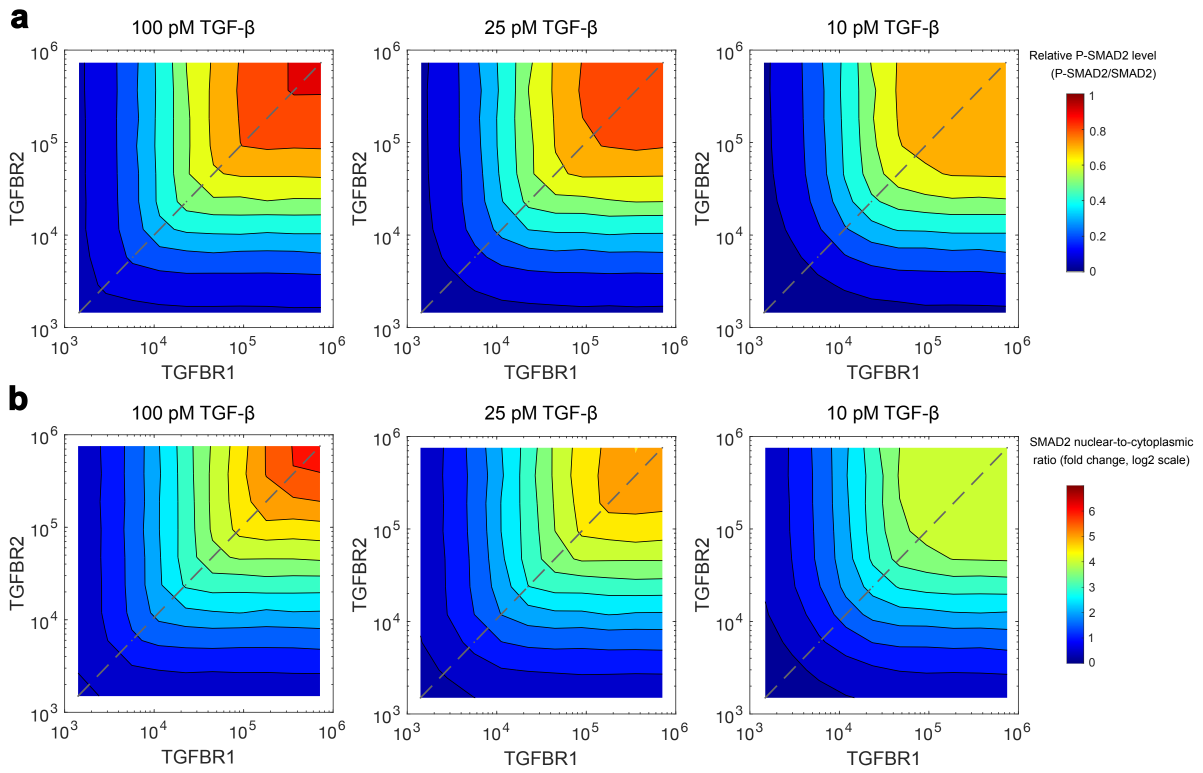

Fig G. Contour landscape of SMAD2 signaling responses in the variation space of TGF-β receptors and SMAD2 under different doses of TGF-β stimulations.

(a) Contour landscape depicting the relative P-SMAD2 level based on combinations of different amounts of TGFBR1 and TGFBR2 with 100 pM, 25 pM, and 10 pM TGF-β stimulations.

(b) Contour landscape showing the fold change in SMAD2 N2C based on combinations of different amounts of TGFBR1 and TGFBR2 with 100 pM, 25 pM, and 10 pM TGF-β stimulation.

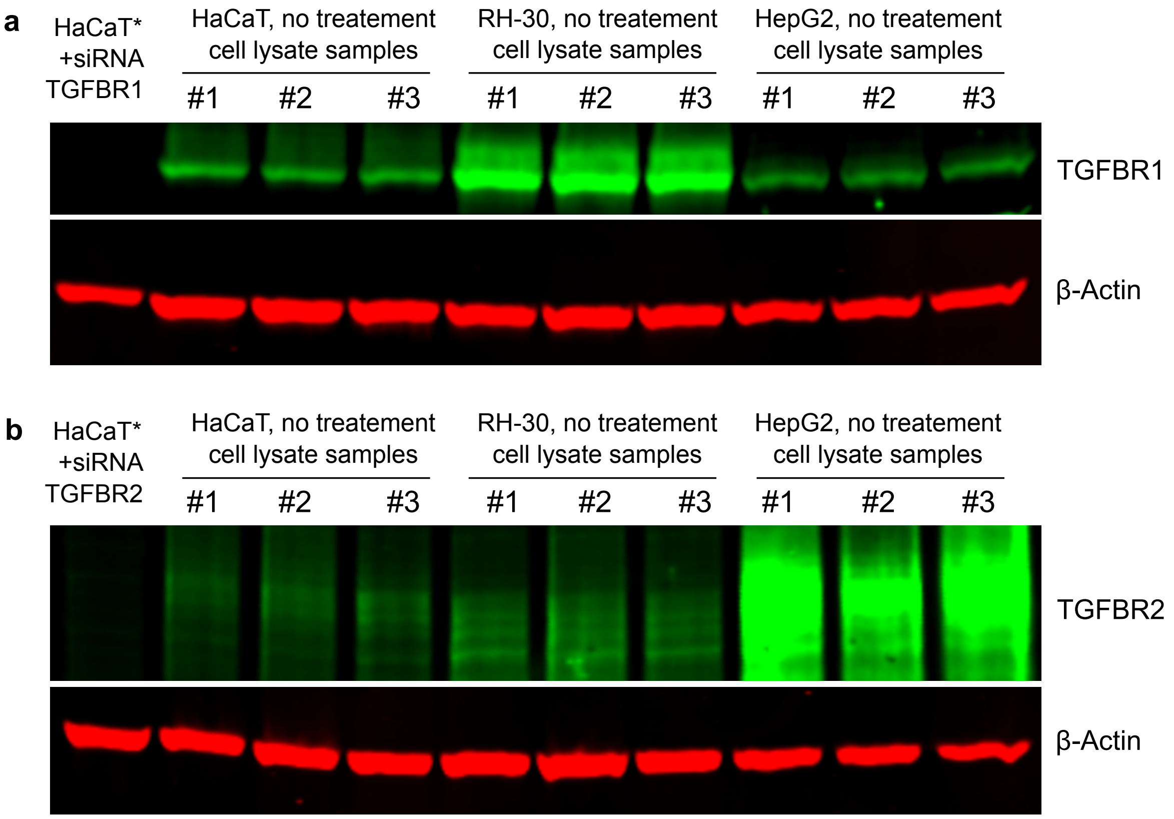

Fig H. TGFBR1 and TGFBR2 protein expression profiles in HaCaT, RH-30 and HepG2 cells

Western blot for measuring relative abundance of TGFBR1 (A) and TGFBR2 (B) in HaCaT, RH-30 and HepG2 cells. The lysate samples from cells (#1, #2, #3) under normal growth conditions (no treatment) were loaded for each cell line. The lysate from HaCaT cells transfected with 100 nM TGFBR1 or TGFBR2 siRNA was loaded as a negative control (the first lane on the left) for the validation of the corresponding antibody.

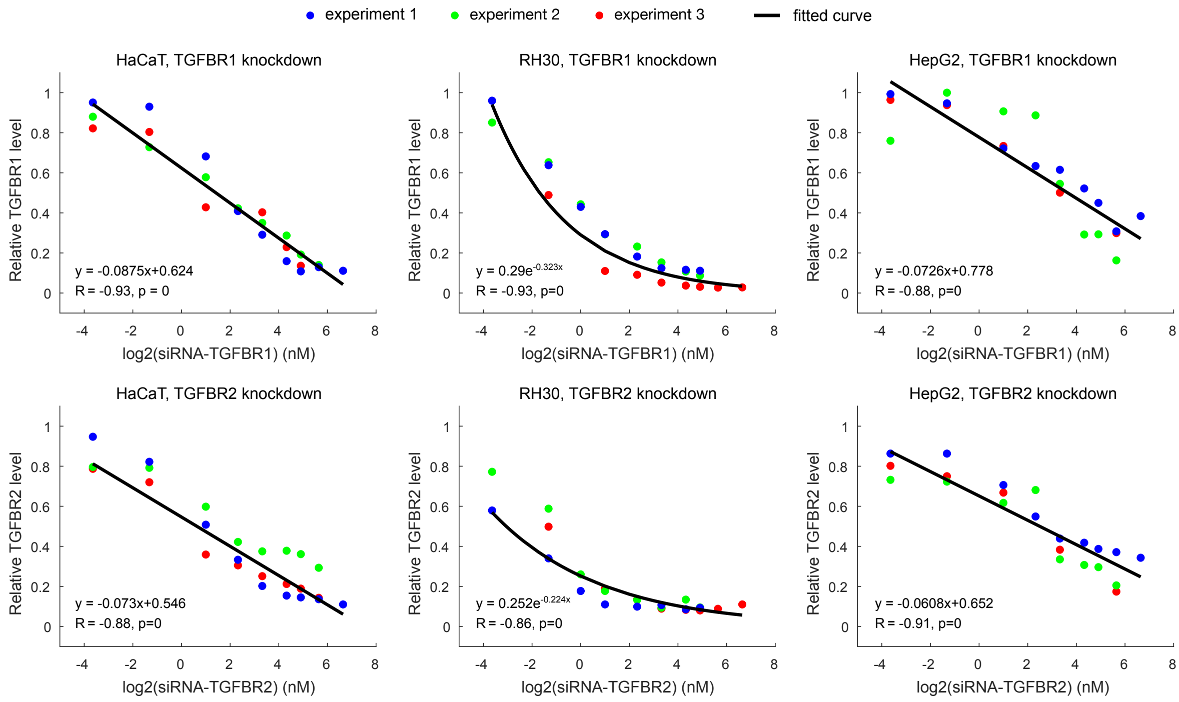

Fig I. Knockdown effect of TGFBR1 and TGFBR2 proteins using different concentrations of siRNAs in HaCaT, RH-30 and HepG2 cells

The relative levels of TGFBR1 and TGFBR2 are normalized to the corresponding TGF-β receptor level of the non-targeting control (ntc) siRNA sample from the same experiment. The siRNA concentrations for TGFBR1 and TGFBR2 are plotted on a log2 scale, and the corresponding relative levels of TGFBR1 or TGFBR2 are displayed. The correlations (R-values and p-values) in the plots were quantified with Pearson correlation coefficients.

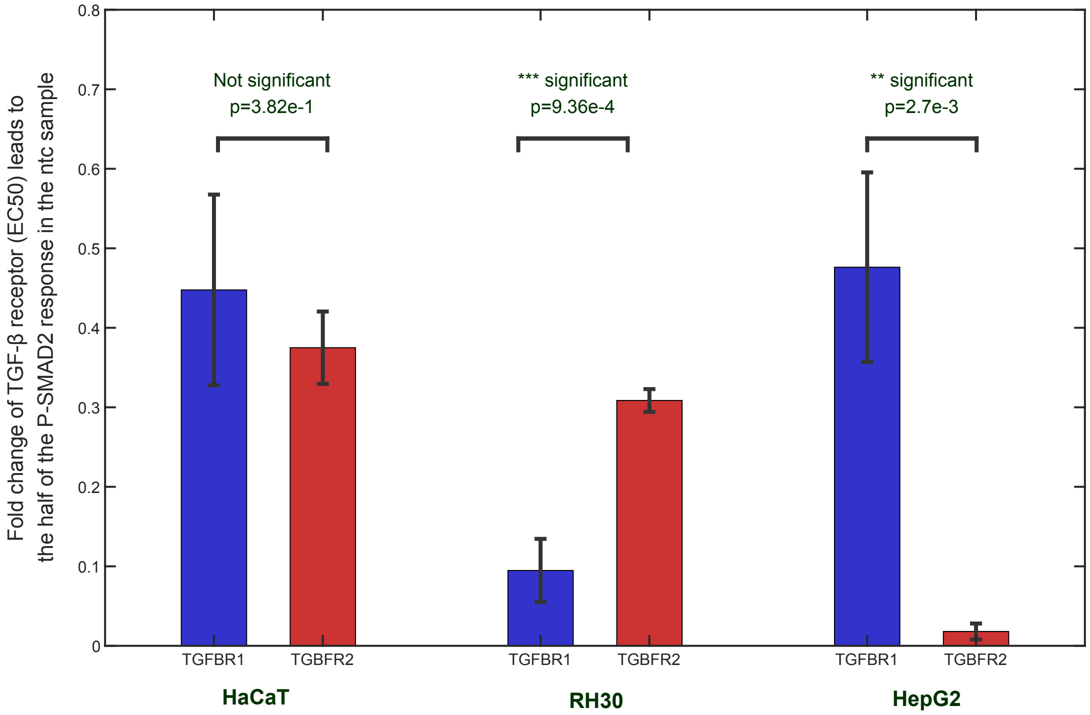

Fig J. Statistical analysis of TGF-β receptor fold-change effects leading to a 50% reduction in the P-Smad2 response compared to that in the non-targeting siRNA control group (EC50) during siRNA knockdown experiments.

The EC50 values were determined by fitting Hill functions to the data shown in Fig 4. Presented here are the average EC50 values along with standard deviations, calculated from three independent experiments. Significance testing was conducted using a two-sample t-test, and significance levels are denoted as follows: ***$p < 0.001$, **$p < 0.01$.

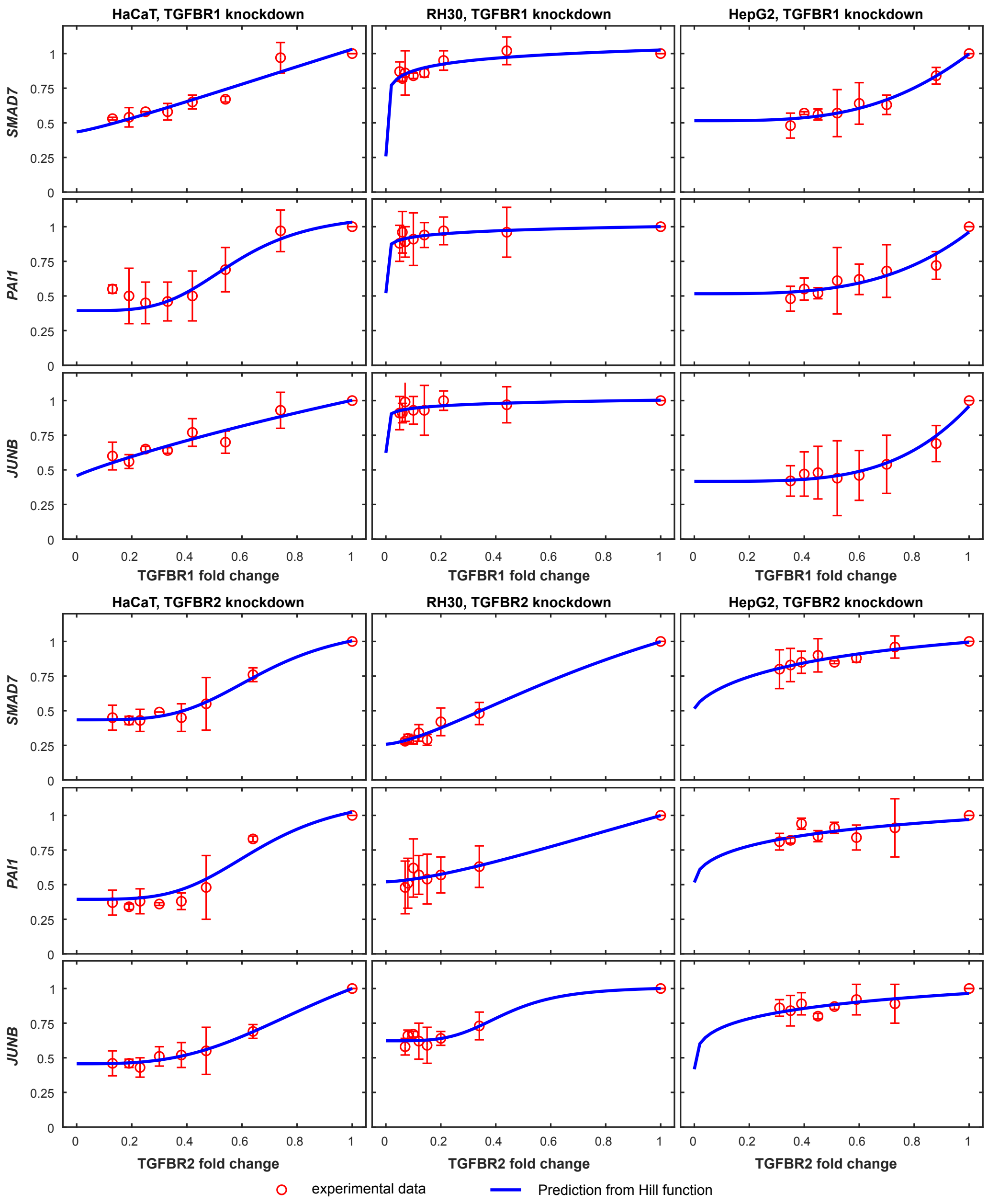

Fig K. Expression of TGF-β targeted genes in responses to the knockdown of TGF-β receptors in HaCaT, RH30 and HepG2 cells

Different levels of each type of TGF-β receptor were achieved by knocking down with various concentrations of siRNA (non-targeting control, 0.4, 2, 5, 10, 20, 30, and 50 nM). The receptor levels were determined using calibration functions shown in Fig I in S1 File. The relative levels of TGF-β targeted genes (*SMAD7*, *PAI1*, and *JUNB*) were measured at 1 hour after 100 pM TGF-β stimulation and normalized to the non-targeting control (ntc) siRNA sample in the same experiment. The error bars represent the standard deviations of the quantified real-time PCR data from three biological replicates.

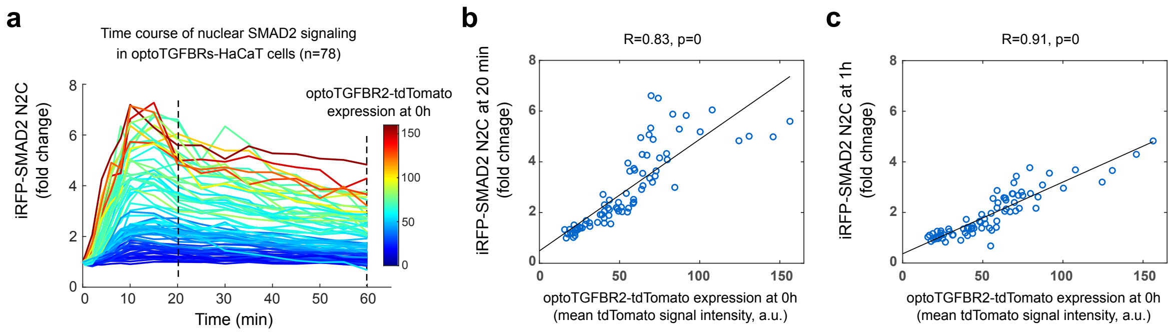

Fig L. Quantification of SMAD2 N2C fold change responses in HaCaT cells expressing similar levels of optoTGFBR1 and optoTGFBR2

(a) Time-course profiles of iRFP-SMAD2 N2C fold change responses in individual optoTGFBRs-HaCaT cells after blue light stimulation.

(b) The plot of iRFP-SMAD2 N2C fold change responses at 20 min versus the amount of optoTGFBR2-tdTomato before blue light stimulation (0 h) in optoTGFBRs-HaCaT cells.

(c) The plot of iRFP-SMAD2 N2C fold change responses at 1 h versus the amount of optoTGFBR2-tdTomato before blue light stimulation (0 h) in optoTGFBRs-HaCaT cells.

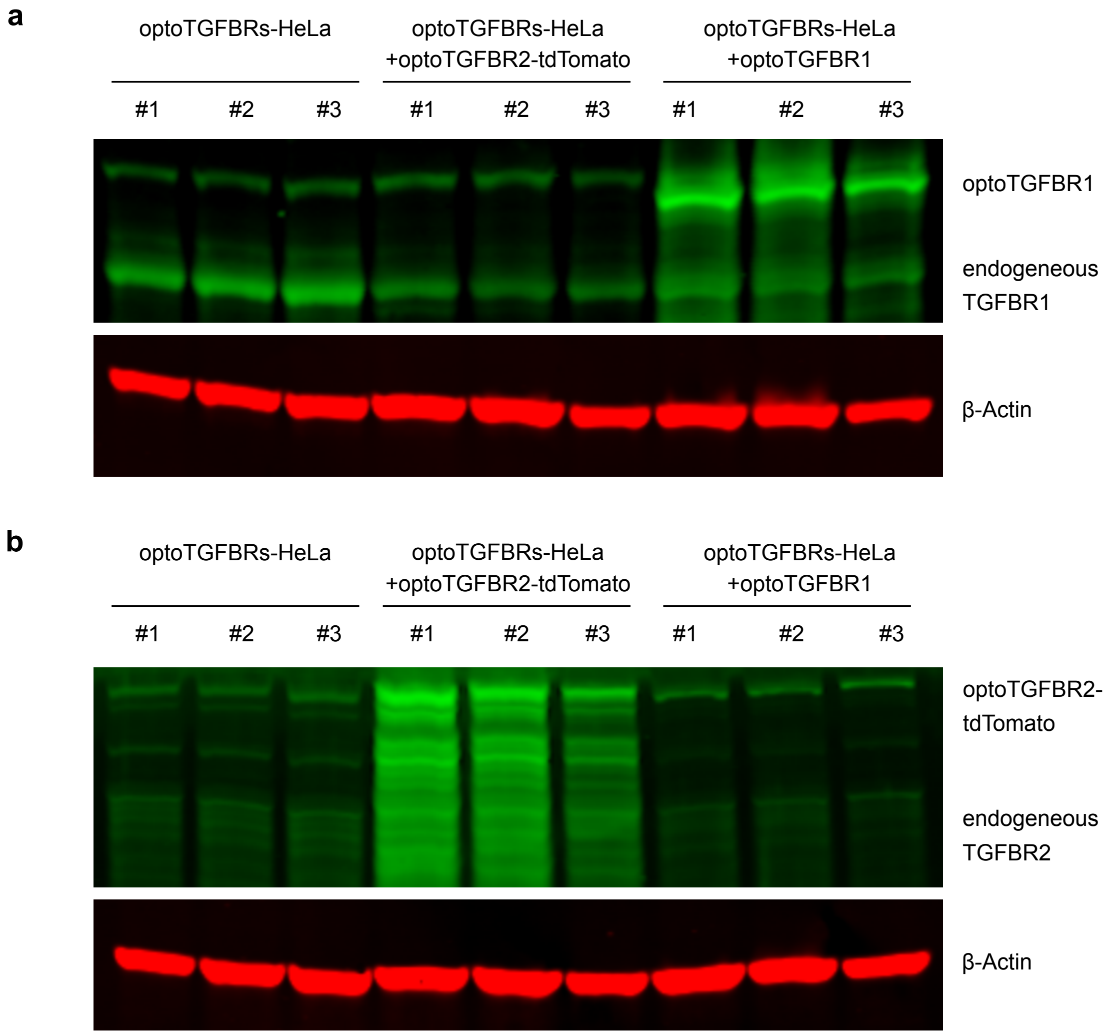

Fig M. Western blot analysis of optoTGFBR1 and optoTGFBR2 protein expression in optoTGFBRs-HeLa cells transfected with optoTGFBR1 or optoTGFBR2 plasmids

Cell lysates were obtained from optoTGFBRs-HeLa cells, optoTGFBRs-HeLa cells transfected with optoTGFBR1 plasmids (optoTGFBRs-HeLa + optoTGFBR1), and optoTGFBRs-HeLa cells transfected with optoTGFBR2-tdTomato plasmids (optoTGFBRs-HeLa + optoTGFBR2-tdTomato). Immunoblotting was performed using antibodies for TGFBR1 (a) and TGFBR2 (b).

References

1. Antebi YE, Linton JM, Klumpe H, Bintu B, Gong M, Su C, et al. Combinatorial Signal Perception in the BMP Pathway. Cell. 2017;170(6):1184-96 e24.
2. Su CJ, Murugan A, Linton JM, Yeluri A, Bois J, Klumpe H, et al. Ligand-receptor promiscuity enables cellular addressing. Cell Syst. 2022;13(5):408-25 e12.
3. Vilar JM, Jansen R, Sander C. Signal processing in the TGF-beta superfamily ligand-receptor network. PLoS Comput Biol. 2006;2(1):e3.
4. Schmierer B, Tournier AL, Bates PA, Hill CS. Mathematical modeling identifies Smad nucleocytoplasmic shuttling as a dynamic signal-interpreting system. Proc Natl Acad Sci U S A. 2008;105(18):6608-13.
5. Zi Z, Feng Z, Chapnick DA, Dahl M, Deng D, Klipp E, et al. Quantitative analysis of transient and sustained transforming growth factor-beta signaling dynamics. Mol Syst Biol. 2011;7:492.
6. Deng D, Zi Z. Absolute Quantification of TGF-beta Signaling Proteins Using Quantitative Western Blot. Methods Mol Biol. 2022;2488:1-12.
7. Wakefield LM, Smith DM, Masui T, Harris CC, Sporn MB. Distribution and modulation of the cellular receptor for transforming growth factor-beta. J Cell Biol. 1987;105(2):965-75.
8. Gabriel L, Stevens Z, Melikian H. Measuring plasma membrane protein endocytic rates by reversible biotinylation. J Vis Exp. 2009;(34).
9. Di Guglielmo GM, Le Roy C, Goodfellow AF, Wrana JL. Distinct endocytic pathways regulate TGF-beta receptor signalling and turnover. Nat Cell Biol. 2003;5(5):410-21.
10. Kao SH, Wang WL, Chen CY, Chang YL, Wu YY, Wang YT, et al. Analysis of Protein Stability by the Cycloheximide Chase Assay. Bio Protoc. 2015;5(1).
11. Zi Z, Klipp E. Constraint-based modeling and kinetic analysis of the Smad dependent TGF-beta signaling pathway. PLoS One. 2007;2(9):e936.
12. Schmierer B, Hill CS. Kinetic analysis of Smad nucleocytoplasmic shuttling reveals a mechanism for transforming growth factor beta-dependent nuclear accumulation of Smads. Mol Cell Biol. 2005;25(22):9845-58.
13. Zi Z. SBML-PET-MPI: a parallel parameter estimation tool for Systems Biology Markup Language based models. Bioinformatics. 2011;27(7):1028-9.
14. Raue A, Kreutz C, Maiwald T, Bachmann J, Schilling M, Klingmuller U, et al. Structural and practical identifiability analysis of partially observed dynamical models by exploiting the profile likelihood. Bioinformatics. 2009;25(15):1923-9.
15. Kaminska B, Wesolowska A, Danilkiewicz M. TGF beta signalling and its role in tumour pathogenesis. Acta Biochim Pol. 2005;52(2):329-37.
16. Wakefield LM, Winokur TS, Hollands RS, Christopherson K, Levinson AD, Sporn MB. Recombinant latent transforming growth factor beta 1 has a longer plasma half-life in rats than active transforming growth factor beta 1, and a different tissue distribution. J Clin Invest. 1990;86(6):1976-84.
