# Supplementary material for: Liebig’s law of the minimum in the TGF-β/SMAD pathway
Source: PLoS Comput Biol. 2024 May 16;20(5):e1012072. doi: 10.1371/journal.pcbi.1012072 (PMC11135686; doi:10.1371/journal.pcbi.1012072)
Supplement: Attachment — Submitted filename: Zi_Response to Reviews in Review Commons.pdf [file pcbi.1012072.s002.pdf]

Full Revision

Review COMMONS

Manuscript number: RC-2023-02073
Corresponding author(s): Zhike, Zi

1. General Statements

We express our gratitude to the reviewers for their time and insightful comments, which have significantly contributed to the enhancement of our manuscript. We believe that the thoughtful critiques and suggestions have substantially improved the overall quality of our work. The changes made in the revised manuscript were highlighted in red. Below, we provide a point-by-point response to each comment, addressing the concerns raised by the reviewers.

2. Point-by-point description of the revisions

Reviewer #1 (Evidence, reproducibility and clarity (Required)):

Summary:

In the current study, Li et al investigated how TGF-beta signaling is controlled by protein abundances. Computational modeling and experiments indicated that the abundance of TGFBR1 and TGFBR2 affects the signaling, and those with lower abundance affect the signaling more, resembling Liebig's law of the minimum. Specifically, they showed that by using multiple cell lines with a different abundance of receptors, modulation of expression of the less abundant receptor impacts the signaling, which is measured by SMAD2 nuclear-to-cytosol ratio and/or relative phospho-SMAD2 level. Also, by using a light-induced interaction system, they showed that the signaling is dependent on the concentration of receptor complex when both receptors are expressed at similar amounts.

Major comments:

Computational predictions support the authors' idea. The computation and the experiments are well-documented. And it would gain substantially if the authors fill the gap between the predictions and the experiments as follows.

In Figure 4, the authors showed that perturbation on receptors with lower expression levels in each cell line changes the phospho-SMAD2 level. Although the data looks consistent with their claim, the result is only qualitative. The authors established a computational model in the former sections, thus it would be of great interest to assess if the experimental results quantitatively match the computational prediction.

Response: The reviewer suggests that our work could benefit from a quantitative comparison between computational predictions and experimental data shown in Figure 4. We appreciate this suggestion. Given the challenges in obtaining precise quantification of TGFBR1 protein due to antibody issues (see the response to comment #2 from reviewer 2), a direct quantitative comparison between model predictions and experimental results is difficult. Our model predictions

about the control principle with Liebig's law of the minimum should be interpreted qualitatively, rather than a strict quantitative law. We have explicitly indicated in the revised manuscript that our siRNA knockdown experiments are to qualitatively test our model predictions.

In Figure 5, the authors computationally predicted that the expression level of receptors is correlated with SMAD2 N2C levels 1 hour after stimulation, and the strength of negative feedback with SMAD2 N2C levels 8 hours after stimulation. Because the authors employed iRFP-SMAD2 system, the prediction could be verified experimentally, at least the prediction on SMAD2 N2C 1 hour after stimulation could be checked. (In a sense, this is partially verified by the data in Figure 7, where both receptors are expressed at similar levels). It would gain substantially if the authors could verify the computational prediction in Figure 6. Since the authors stated in the introduction that "The same TGF-beta ligand can initiate different signaling responses depending on the cellular context, but the underlying control principle remains unclear...Together, these results revealed an effect of the minimum control in the TGF-beta pathway, which may be an important principle of control in signaling pathways with context-dependent outputs.", experimental verification of the prediction done in Figures 4-6 will be very important. Or the authors should stress that these points are only predicted by computational models.

Response: The reviewer recommends verifying the model predictions in Figure 6 experimentally, particularly regarding SMAD2 N2C levels 1 hour after stimulation. We appreciate this valuable suggestion, which was also raised by reviewer 2. In response, we conducted experiments as recommended by reviewer #2, in which imbalanced expression of TGFBR1 and TGFBR2 was achieved by transfecting optoTGFBR1 or optoTGFBR2 plasmids into optoTGFBRs-HeLa cells, which initially expressed similar levels of both receptors. Western blot analysis confirmed the desired imbalance (Figure S13).

Consistent with the model predictions (Figure 6), the strong correlation between SMAD2 N2C fold change response at 1h and optoTGFBR2-tdTomato expression levels persisted in single cells when optoTGFBR1 was overexpressed (Figure 8A). Conversely, the high correlation between nuclear SMAD2 signaling and optoTGFBR2-tdTomato expression levels vanished at single cell level when optoTGFBR2 was overexpressed (Figure 8B). These experimental results validate our model predictions, confirming that the SMAD2 signaling is determined by the low abundance TGF-beta receptor in single cells. Incorporating these experimental validations enhances the quantitative support for our model predictions and clarifies the relationship between TGF-beta receptor abundance and signaling outcomes in single cells.

As written in the below "Significance" section, the result is, in a sense, obvious. It should be stated that because the study utilized a slightly high concentration of TGF-beta in the experiments, it might be natural that the low-abundance receptor becomes a bottleneck of the signaling. It would gain to assess how receptor abundance affects signaling with the stimulation of lower concentrations of TGF-beta, or to examine the computational model if the low abundance of a receptor becomes a bottleneck of signaling because of saturation. Also, it is highly recommended to discuss the physiological implication of the current study, taking into account the experimental conditions used.

Review COMMONS

Response: We appreciate the reviewer's insightful comments regarding the concentration of TGF-beta used in our experiments and the potential influence on the model predictions. In our experiments and model simulations, we utilized 100 pM TGF-beta, equivalent to 2.5 ng/mL (not 4.4 ng/mL as calculated by the reviewer). This concentration is a widely used dose in TGF-beta signaling studies. The reviewer's suggestion to explore how varying TGF-beta concentrations might influence the minimum control concept prompted us to extend our computational simulations. We used the extended model to perform simulations with lower TGF-beta concentrations (25 pM, equivalent to 0.625 ng/mL, and 10 pM, equivalent to 0.25 ng/mL). The results, depicted in Figure S7 of the revised manuscript, reaffirm that even at lower TGF-beta stimulations, a low abundance of a TGF-beta receptor acts as a bottleneck for SMAD2 signaling.

Following the reviewer's suggestion, we have incorporated additional paragraphs to discuss the physiological implications and potential limitations of our study (Page 16-17 in the Main text).

It is pertinent to note that while the concept of TGF-beta signaling response being dictated by the minimum abundance of TGF-beta receptors may seem intuitive or even obvious, theoretical and experimental validations are crucial. As demonstrated in Figure S1B, our new simulation results from the minimal model illustrate similar response profiles when a high binding affinity (K1) is set for ligand-receptor interactions (Figure S1A). However, with a small binding affinity (K1), the minimal model indicates that TGF-beta signal response remains proportional to the product of TGFBR1 and TGFBR2 abundance and can be sensitive to the change of high abundance receptor in some region (Figure S1B). This highlights that the observed response patterns aligning with Liebig's law of the minimum depend on the binding affinity of ligand-receptor interactions in our minimal model. **Consequently, the intuitive idea about Liebig's law of the minimum is not necessarily true theoretically.** Moreover, given the non-linearity of the TGF-beta network, this complexity introduces an additional layer of uncertainty regarding the applicability of the minimum control principle to TGF-beta responses. This uncertainty led us to develop an extended model, with parameter values either experimentally measured or estimated from time course experimental data. The extended model predicted a similar minimum control principle at the TGF-beta receptor level, inspiring us to validate this prediction through diverse experiments. While we acknowledge the intuitive nature of our findings, we believe it is important for the field to prove this expectation, as emphasized by reviewer 4.

Reviewer #1 (Significance (Required)):

TGF-beta signaling is one of the most rigorously studied pathways both computationally and experimentally. As written in the introduction of the manuscript, it is still unknown how the variability of responses arises not only between cell types but also differences among cells of single cell type. Studies showed that protein abundance accounts at least partly for a source of cell variability in TGF-beta signaling. While former studies examined the variability in SMAD protein abundance, the uniqueness of this study is that it focused on the abundance of TGF-beta receptors.

Given that both TGFBR1 and TGFBR2 are involved in the signaling, however, it's not difficult to imagine that a less abundant receptor affects the signaling more than the other, and serves as a bottleneck for the signaling. Specifically, because a slightly high concentration (100pM = 4.4 ng/mL of TGF-beta; other studies used much lower conc., e.g. 0, 0.03, 0.04, 0.07, and 2.4 ng/mL in Frick et al, PNAS, 2017, and 0, 1, 2.5, 5, 25, and 100 pM in Strasen et al, Mol Syst Biol, 2017) is used throughout the experiments to check cell-cell variability and the effect of receptor abundance in the current study, the formation of the receptor-ligand complex may be quite fast and be saturated at the level where the receptor with lower abundance is exhausted. In the reviewer's humble opinion, the authors' statement that this is Liebig's law of the minimum sounds a bit exaggerated.

Nevertheless, the study is of some value because it utilized both computational and experimental analysis to show it is indeed the case. Of note, the current study showed that the variability in the different proteins leads to the variability in different time points, namely, the variability in the receptor abundance leads to the variability 1 hour after stimulation, while that in negative feedback strength leads to the variability 8 hours after stimulation. If the authors fill a small gap between their computational analysis and experimental verification, the study will be of interest to the specialist in the field.

Response: We are grateful for the valuable feedback provided by the reviewer. The concerns related to the TGF-beta dose have been thoroughly addressed in our responses to previous comments. Regarding the observation that the term "Liebig's law of the minimum" may sound a bit exaggerated, we acknowledge this consideration. We have refined the title to "Liebig's Law of the Minimum in the TGF-β/SMAD Pathway," specifying its relevance to SMAD signaling exclusively, as non-SMAD signaling was not within the scope of this study. We appreciate the reviewer's constructive feedback and hope these adjustments enhance the specificity and accuracy of our manuscript.

Reviewer #2 (Evidence, reproducibility and clarity (Required)):

Li et al. present an interesting and intuitive concept for the sensitivity and heterogeneity of biological networks: When two or more proteins form a functional complex, it is the limiting component with the lowest concentration that is most sensitive to perturbations and whose fluctuations dictate cell-to-cell variability of complex function. The authors apply this concept to the TGFb pathway and discuss sensitivity of SMAD signaling towards TGFb receptor I and II fluctuations. The paper is clearly written and convincing, but some improvements in the experimental validation would be beneficial as detailed further below.

1) The authors claim that the ratio of TGFb receptor I and II is very different across cell lines (Fig. 1) and use this observation for the validation of their model in Fig. 4. However, the relative expression TGFb receptor levels are purely based on RNAseq data which does not necessarily imply similar behavior at the protein level, especially on the cell surface. To address this issue, the authors should ideally provide absolute Western blot measurements of TGFbRI at the protein

level to complement their absolute quantification of TGFbRII (Fig. S2). At the very least they should show that the observed relative expression levels of TGFbRI and II at the protein level (Figure S7) are correlated to differences in RNA levels (Fig. 1) using protein quantification. They should also confirm that similar receptor ratios for these receptors at the RNA level are observed in other published RNAseq datasets of the same cell lines (e.g., ENCODE for HepG2 and published RNAseq studies in HaCaT). Furthermore, they might take into account published mass spec datasets for quantifications of TGFbR protein levels.

Response: We appreciate the reviewer's thorough evaluation and constructive suggestions.

(A) Absolute quantification of TGFBR1: We acknowledge the importance of obtaining absolute quantification of TGFBR1 protein similar as what we have done for TGFBR2 protein (Figure S2). Despite significant efforts, our attempts to achieve this were hindered by challenges with available TGFBR1 antibodies and recombinant TGFBR1 proteins. Many commercial antibodies failed negative controls with TGFBR1 knockdown samples, while others validated TGFBR1 antibodies could not recognize the available recombinant TGFBR1 protein standards.

Although many mass spectrometry proteomics data available for different cell lines, it is difficult to convert these MS quantitative values to absolute protein abundance as mentioned in a recent publication (Nusinow *et al.*,bioRxiv 2020.02.03.932384): "*Importantly, these values are all relative values to the other values for that same protein and not absolute values. This means that comparing the levels of different proteins to each other without using something like a correlation to standardize values won't produce meaningful results.*"

We share the reviewer's concern and fully agree that obtaining this absolute quantification is crucial. However, at the present stage, technical limitations prevent us from providing this information for TGFBR1. We commit to pursuing this aspect when feasible in the future.

(B) Validation of relative TGF-beta receptor expression ratios: Following the reviewer's suggestion, we conducted additional analyses to validate the relative expression ratios of TGFBR1 and TGFBR2 using different RNA-Seq databases. The results, presented in Table S1, demonstrate consistent imbalances in TGFBR1-to-TGFBR2 ratios across HepG2 and RH30 cell lines from various data sources, reinforcing the reliability of our observations.

(C) Correlation between RNA and protein expression: We appreciate the reviewer highlighting the challenges associated with correlating RNA and protein expression. Indeed, the correlations between RNA and protein levels vary widely, and direct comparisons can be challenging. To address this, we referenced a recent study (Nusinow et al., Cell 2020, 180:387), which reported that the protein data of TGFBR1 and TGFBR2 were highly correlated with the corresponding RNA data from the same cell line (Spearman's correlation: 0.672 for TGFBR1, 0.771 for TGFBR2) based on quantitative proteomics and RNA expression data from 375 cancer cell lines.

2) Figure 4: To better judge the reproducibility of the knockdown titration, it would be good to show the different siRNA concentrations as a color code- Alternatively, TGFBR expression could be plotted as a function of the siRNA concentration in a Supplemental Figure, showing the effects of individual replicates.

Response: We thank the reviewer for the suggestion to enhance the clarity of the knockdown titration data. In response, we have now presented the quantified experimental data from three replicates with different colors in Figure 4. Additionally, we have created Figure S9 that plots the expression levels of relative TGFBR1 and TGFBR2 as a function of siRNA concentration, providing a more detailed view of the effects across individual replicates.

3) The simulations in Figs. 5 and 6 show that SMAD signaling fluctuations are mainly determined by cell-to-cell variability of receptor levels when using the SMAD nucleocytoplasmic ratio as a readout, and this is especially true for early time points. For downstream cellular responses, the absolute concentration of phosphorylated SMAD (complexes) in the nucleus is likely more relevant. Based on the authors work and evidence from the literature, I expect that this quantity will likely be heavily be influenced by receptor levels as well, but fluctuations in SMAD expression will play an important role as well. The authors should discuss this issue, and clarify that normalized quantities like SMAD N2C and pSMAD/SMAD mostly characterize receptor-level fluctuations while filtering SMAD fluctuations.

Response: We acknowledge the importance of discussing the relevance of different readouts in our study. In the revised manuscript, we have incorporated a discussion addressing this issue. Specifically, we highlight that while the SMAD nucleocytoplasmic ratio is sensitive to cell-to-cell variability in low abundance receptor levels, the absolute concentration of phosphorylated SMAD in the nucleus may be more relevant for downstream cellular responses (e.g.: gene expression). We have cited the work by Lucarelli et al, which demonstrated that variations in SMAD abundance could modulate the balance of different SMAD complexes, thereby regulating heterogeneous gene expression in diverse cell types (Lucarelli et al., Cell Systems 2018).

4) The single-cell measurements in Fig. 7 are interesting, but can only partially be seen as a direct validation of the model predictions, as it seems expected that varying the total input by introducing co-fluctuations in both receptors heavily influence the SMAD level. Wouldn't it be possible to design more specific validation experiments, in which the receptor co-expression construct (Fig. 7C) is used for baseline optoTGFBR expression and combined with an individual expression construct for one of the opto-receptors? This way, the authors could establish different regimes, in which one of the two receptors becomes dominant, and the impact fluctuations could be analyzed in a larger receptor expression space. Of course, a full validation of all possible scenarios is not necessary, but it would, for instance, be valuable to see whether the strong dependency of SMAD signaling of TGFBR2 levels vanishes when TGFBR2 is expressed at a higher level than TGFBR1.

Response: We appreciate the insightful comments and suggestions provided by the reviewer. Based on these recommendations, we have conducted additional experiments to further validate our model predictions. Reviewer 1 also raised this point, we quote our aforementioned response here: "consistent with the model predictions (Figure 6), the strong correlation between SMAD2 N2C fold change response at 1h and optoTGFBR2-tdTomato expression levels persisted in single cells when optoTGFBR1 was overexpressed (Figure 8A). Conversely, the high correlation between nuclear SMAD2 signaling and optoTGFBR2 expression levels vanished at single cell

level when optoTGFBR2 was overexpressed (Figure 8B). These experimental results validate our model predictions, confirming that the SMAD2 signaling is determined by the low abundance TGF-beta receptor in single cells. Incorporating these experimental validations enhances the quantitative support for our model predictions and clarifies the relationship between TGF-beta receptor abundance and signaling outcomes in single cells."

Referees cross-commenting

Comments from R2: I agree with most comments of the other reviewers, and highlight the most important overlaps with my comments below.

I agree with R1 that the model validation in Fig. 7 is incomplete and think that this will be a key point to improve the quality of the manuscript (see also my reviewer comment 4)

In line with R3 and R4, I think that the SMAD N/C simulations do not necessarily imply effects on TGFb target gene expression, cell fate decisions or human pathologies. The significance of the results for cellular behavior should be discussed (see also my comment 3)

Response: We are grateful for the reviewer's thoughtful comments. These comments have been now addressed (see our responses to the corresponding comments).

Reviewer #2 (Significance (Required)):

The manuscript presents an interesting and intuitive concept for the sensitivity and heterogeneity of biological networks. The authors apply this concept to the TGFb pathway and discuss sensitivity of SMAD signaling towards TGFb receptor I and II fluctuations.

Reviewer #3 (Evidence, reproducibility and clarity (Required)):

Summary:

This is an interesting study that examines the output of the TGF-Beta pathway and how abundance/dosage can determine the signaling response in single cells across multiple cell types. The study is primarily mathematical. The focus is on the Type 1 and 2 TGF-Beta receptors driving nuclear SMAD2 expression. The authors observe that SMAD2 phosphorylation is sensitive to variations in the lower levels of either receptor but robust at variations of high abundance of the receptor reflected through SiRNA experiments shown in Figure 4. Their conclusion is that the feature is consistent with Liebig's law of the minimum- where in this case- a low abundance of the receptor serves as the rate-limiting step in signaling for this pathway.

Major comments:
- While the data as presented are interesting, it is unclear as to whether the abundance regulates biological function. SMAD2 phosphorylation is shown with some nuclear translocation. However, TGF-Beta target gene activation is not shown, and this needs to be completed.

Response: We appreciate the reviewer's constructive comment. We have conducted new experiments and included quantitative real-time PCR data in the revised manuscript to evaluate the impact of TGFBR1 and TGFBR2 knockdown on the expression of TGF-beta target genes, such as *SMAD7*, *PAI1*, and *JUNB*. The results, presented in Figure S11, demonstrate differential sensitivity of these genes to the downregulation of TGFBR1 and TGFBR2 in various cell lines (HaCaT, HepG2, and RH30). Specifically, the expression of *SMAD7*, *PAI1*, and *JUNB* is sensitive to TGFBR2 knockdown in RH30 cells, while it is sensitive to TGFBR1 knockdown in HepG2 cells. HaCaT cells, expressing similar levels of both receptors, show comparable sensitivities to reductions in both TGFBR1 and TGFBR2. These findings provide additional insights into the regulatory role of TGF-beta receptor abundance on downstream target gene activation, complementing our study's focus on SMAD2 phosphorylation and nuclear translocation.

- In addition, it is unclear as to what happens to SMAD3 and SMAD4 which are expressed endogenously in this setting. How are these other TGF-Beta signaling molecules addressed by these observations?

Response: Thank you for bringing up this important point. In our study, the expression levels of endogenous SMAD2 and SMAD4 were found to be similar across HaCaT, RH30, and HepG2 cells. However, SMAD3 expression was notably lower in RH30 and HepG2 compared to HaCaT cells. The central conclusion of our study is based on the observed common control principle, which hinges on the relative expression levels of TGFBR1 and TGFBR2. Consequently, the applicability of this principle is more pertinent when comparing signal responses within the same cell type.

We acknowledge the relevance of endogenous SMAD proteins, and in the revised manuscript, we have expanded our discussion on how differences in SMAD protein expression levels and potential mutations (page 16 in main text), as observed in certain cancers, could influence the formation of homo- and hetero-oligomeric SMAD complexes. These considerations contribute to a more comprehensive understanding of downstream gene expression responses, as discussed in the work of Lucarelli et al. (Cell Systems 2018).

-Specific biological readouts- cell differentiation etc. are not examined and would need to be provided and discussed. Therefore, the claims put forward while interesting require additional experiments examining SMAD2 target gene activation and biological readouts.

Response: We appreciate this valuable suggestion. While we acknowledge the importance of exploring long-term biological responses, including cell differentiation, it is crucial to note that specific biological readouts are not solely dependent on SMAD signaling; they also involve other non-SMAD signaling pathways. Additionally, these responses are highly cell type-specific. Undertaking extensive investigations into these responses would extend beyond the current scope of our work. Nevertheless, we have discussed this topic in the revised manuscript (page 16 in main text).

Following the reviewers' suggestion on examining TGF-beta target genes, we have performed experiments examining the expression of *SMAD7*, *PAI1*, and *JUNB* with respect to the changes

of TGFBR1 and TGFBR2, respectively (see our response to the first major comment of this reviewer).

- Lastly, statistical analyses are not provided and would need to be provided. For instance, in Figure 4, how many experiments were replicated and statistical analysis performed for this Figure?

Response: In addressing this concern, we conducted three siRNA knockdown titration experiments for each cell line, as detailed in the figure legend. Due to batch effects, different percentages of TGF-beta receptors were knocked down in different experiments using the same concentration of siRNA. To transparently present the data, we utilized a scatter plot. Following the suggestion from reviewer 2, we have further enhanced the clarity of our data presentation by labeling the results of different experiments with a color code. In addition, we have performed statistical analysis of TGF-β receptor fold-change effects leading to a 50% reduction in the P-Smad2 response compared to that in the non-targeting siRNA control group (EC50) during siRNA knockdown experiments (Figure S10). The results of this analysis unveil significant differences in the sensitivities of pSMAD2 responses to variations in TGFBR1 and TGFBR2 within RH30 and HepG2 cells.

Reviewer #3 (Significance (Required)):

- Conceptually this is an important study because dosage is a prominent issue in TGF-Beta signaling. For instance, in my field of expertise- mouse models of TGF-beta signaling e.g. SMAD2 knockouts- the cancer phenotypes are evident in haploid animals. Yet how and why dosage plays such a large role in tumorigenesis remains unclear.

Response: We sincerely appreciate your recognition of the conceptual importance of our study in addressing the dosage-related complexities of TGF-beta signaling. Your insights into dosage effects in mouse models, particularly in haploid animals, highlight the relevance of our work underlying tumorigenesis. We have incorporated relevant citations and expanded our discussion in the revised manuscript, providing additional context to the importance of dosage in tumorigenesis (page 18 in main text).

Reviewer #4 (Evidence, reproducibility and clarity (Required)):

Summary: In this study, Li and co-workers combined computational modeling and experimental analysis to study the dependence of the output of the TGF-beta pathway on the abundance of signaling molecules in the pathway, mainly the most upstream regulators of SMAD2, TGFbeta type I and type II receptors. They showed by a combination of biochemical studies (mainly pSmad2 WB and type I/II receptor expression profiling) in HaCaT and HeLa cells as well as stable optogenetical receptor variants expressed by those cell lines, that TGF-beta receptor abundance influences signaling outputs using the concept of Liebigs law of the minimum, meaning that the output-modifying factor is the signaling protein that is most limited, to determine signaling responses across cell types and in single cells.

Major comments:

The study is very interesting, the combination of biochemistry and computational modeling to better understand the compexity of the TGFbeta pathway is very much required in the field and should stimulate others to further expand this approach.

Response: Thank you for the positive evaluation of this work.

However, the authors must further explain that the model depicted here to explain pathway kinetics and dynamics lacks multiple crossroads and feedbacks and is until now oversimplified in the manuscript. They have mentioned receptor internalization and recycling, nuclear import and export of SMAD protein, and the feedback regulations e.g. by SMADs regulating receptor expression. Beyond, there is non- SMAD signaling (Derynck et al.; SMAD Linker regulation, deRobertis et al.), different receptor oligomerization modes (Ehrlich/Henis et al.) and heteromeric receptor complexes of TGFbeta receptors known (Hill et al.), that further diversify beyond these mentioned mechanisms. It is understandable that the mathematical model cannot include those considerations to date, however, they must be further explained and commented on to allow that this model can be expanded in the future.

Response: We acknowledge that there are multiple crossroads and feedbacks that exist in the TGF-beta signaling pathway that have not been explicitly incorporated into our model. We appreciate the reviewer's understanding that current model cannot include these considerations and his/her suggestions for potential future extensions. In the revised manuscript, we have mentioned one of the limitations of our model: non-Smad signaling and crosstalk with other signaling pathways were not considered for simplicity. We have also discussed how to expand this model by including these regulations when more quantitative data are available in the future (page 16-17 in main text).

A myriad of research labs focus on these intricate fine tuning ot the TGFbeta pathway by those mechanisms which makes the difference between "good" TGFbeta signaling and "bad" TGFbeta signaling in different context and this complexity must be acknowledged by more introduction and discussion.

Response: In the revised manuscript, we have added an introduction and discussion about the dual role of TGF-beta signaling (page 4 and page 18 in main text).

The model here will be important to explain

A: the mode of heterooligomeric TGFbeta/BMP receptor assemblies as e.g. found in pathological conditions and

B: Can maybe explain the formation of mixed SMAD complexes as activated by lateral signaling comprising TGFbeta and BMP receptors once one receptor is of lower abundance to form a high affinity complex.

It is therefore required to comment on these aspects at multiple points in the manuscript.

It is very important that the visual model used in this manuscript depicts on the possibility, that a TGFbeta type I receptor can team up with e.g. another TGFbeta type I receptor together with two TGFbeta type II receptors but also with an activin type II receptor or that a BMP type I receptor (e.g. ALK1) can form heterooligomeric complexes with ALK5 (TGFbeta type I).

Response: Thank you for this comment. We cited the relevant work (Ramachandran et al, eLife 2018; Szilagyi et al, BMC Biology 2022) and added a discussion about the complexity of the mode of heterooligomeric TGFbeta/BMP receptor assemblies and its effect on the induction of mixed SMAD complexes (page 17 in the main text).

While the use of optogenetical TGFbeta receptor biosensors is highly interesting, their mode of oligomerization is not yet fully described. It is not known if those biosensors behave like wt receptors in terms of oligomerization and ligand binding. This should be mentioned somewehere. For this reason, the authors should also consider to draw the TGFbeta receptor complex in the cartoons with more detail towards the heterooligomeric assembly that is standard to the field.

Response: The reviewer is correct that the optogenetic TGF-beta receptors might behave differently from the natural TGF-beta receptor system in terms of ligand binding. We have added this point in the Discussion part to highlight the potential difference between the optogenetic TGF-beta systems and the wild-type system (page 16 in the main text).

While the general finding is not surprising (manipulating the receptor with the lowest abundancy has the biggest impact on signaling output) the methods and models used here are very important to the field to proof that this expectation is actually true and can be experimentally addressed by a combination of bioinformatics and biochemistry. The model developed will be valuable to expand to much more complex and interesting questions in TGFbeta signaling and possibly also BMP signaling e.g. in pathological context (see below).

Minor comments:

The authors should discuss their findings in the context of:

1. non-Smad signaling outputs (similar or different to the observations on pSMAD2)

2. What do these findings mean for e.g. human pathologies, where type I or type II receptor expression is altered?

3. Can those findings integrate into the "switch" in TGFbeta signaling?

4. How do these findings translate towards BMP SMAD 1/5/9 signaling?

Response: First, we sincerely appreciate the reviewer's recognition that our work is very important to the field in proving that manipulating the receptor with the lowest abundance has the biggest impact on signaling output. The reviewer's suggestions about discussing our work in the context of non-Smad signaling, BMP SMAD1/5/9 branch, and the relevance to the dual role of

TGF-beta signaling are all constructive. We have incorporated these suggestions and discussed them in the revised manuscript (page 17 in the main text).

Reviewer #4 (Significance (Required)):

The manuscript is novel and interesting, partiular the combination of bioinformatical and biochemical approaches. The use of optogenetics is state-of-art while some more care should be given to interpretation of results with optogenetical TGfbeta receptor biosensors, is is not known if they really behave similar in terms of receptor oligomerization and signaling. Also it is not shown how their interactome in terms of effector proteins looks like that can potentially influence SMAD signaling output (e.g. Phosphathases to SMADs known to interact with wt receptors).

The models drawn need to depict more accurately on the nature of type I and type II receptor complexes (heterotetrameric) and high affinity towards the ligand. The current versions are too oversimplified at this stage. The pathway crosstalks and feedbacks need to be more visible, in order for non experts to not draw too simple conclusions from the visual representations presented in this MS. Particularly the work by Hill and co-workers on receptor oligomerization and SMAD shuttling and feedback need to be included.

Overall, the manuscript is very significant to the field.

Response: We would like to thank the reviewer again for his/her positive evaluation of the novelty and significance of our work. We have taken the reviewer's comments into consideration and made revisions to the manuscript. We now provide more information on the limitations of our current model and the optogenetic TGF-beta receptor biosensors in the Discussion section. We have also included more details about the receptor complex nature and the high affinity towards the ligand. The ligand receptor complex in the model is now drawn as heterotetrametric complex (1 ligand dimer with two TGFBR1s and two TGFBR2s). Additionally, we have incorporated information about pathway crosstalks and feedbacks, giving a more comprehensive view for non-experts. The work by Hill and co-workers on receptor oligomerization, SMAD shuttling, and feedback has been included in the revised manuscript to provide a more complete and accurate representation of the current knowledge in the field.
