# Supplementary material for: Liebig’s law of the minimum in the TGF-β/SMAD pathway
Source: PLoS Comput Biol. 2024 May 16;20(5):e1012072. doi: 10.1371/journal.pcbi.1012072 (PMC11135686; doi:10.1371/journal.pcbi.1012072)
Supplement: Peer review history

# Author comment: Author response to previous submission

**DOI:** 10.1371/journal.pcbi.1012072.r001

14 Feb 2024

Attachment Submitted filename: Zi_Response to Reviews in Review Commons.pdf

# Editor report: Decision Letter 0

**DOI:** 10.1371/journal.pcbi.1012072.r002

**Authors:** Mark Alber, James R. Faeder

11 Apr 2024

Dear Dr. Zi,

We are pleased to inform you that your manuscript 'Liebig’s law of the minimum in the TGF-β/SMAD pathway' has been provisionally accepted for publication in PLOS Computational Biology.

Before your manuscript can be formally accepted you will need to complete some formatting changes, which you will receive in a follow up email. A member of our team will be in touch with a set of requests.

Please note that your manuscript will not be scheduled for publication until you have made the required changes, so a swift response is appreciated.

IMPORTANT: The editorial review process is now complete. PLOS will only permit corrections to spelling, formatting or significant scientific errors from this point onwards. Requests for major changes, or any which affect the scientific understanding of your work, will cause delays to the publication date of your manuscript.

Should you, your institution's press office or the journal office choose to press release your paper, you will automatically be opted out of early publication. We ask that you notify us now if you or your institution is planning to press release the article. All press must be co-ordinated with PLOS.

Thank you again for supporting Open Access publishing; we are looking forward to publishing your work in PLOS Computational Biology. 

Best regards,

James R. Faeder

Academic Editor

PLOS Computational Biology

Mark Alber

Section Editor

PLOS Computational Biology

***********************************************************

# Editor report: Acceptance letter

**DOI:** 10.1371/journal.pcbi.1012072.r003

**Authors:** Mark Alber, James R. Faeder

13 May 2024

PCOMPBIOL-D-24-00273 

Liebig’s law of the minimum in the TGF-β/SMAD pathway

Dear Dr Zi,

I am pleased to inform you that your manuscript has been formally accepted for publication in PLOS Computational Biology. Your manuscript is now with our production department and you will be notified of the publication date in due course.

The corresponding author will soon be receiving a typeset proof for review, to ensure errors have not been introduced during production. Please review the PDF proof of your manuscript carefully, as this is the last chance to correct any errors. Please note that major changes, or those which affect the scientific understanding of the work, will likely cause delays to the publication date of your manuscript. 

Soon after your final files are uploaded, unless you have opted out, the early version of your manuscript will be published online. The date of the early version will be your article's publication date. The final article will be published to the same URL, and all versions of the paper will be accessible to readers.

Thank you again for supporting PLOS Computational Biology and open-access publishing. We are looking forward to publishing your work! 

With kind regards,

Anita Estes

PLOS Computational Biology | Carlyle House, Carlyle Road, Cambridge CB4 3DN | United Kingdom ploscompbiol@plos.org | Phone +44 (0) 1223-442824 | ploscompbiol.org | @PLOSCompBiol
